# Spatial lipidomics reveals brain region-specific changes of sulfatides in an experimental MPTP Parkinson’s disease primate model

**DOI:** 10.1038/s41531-023-00558-1

**Published:** 2023-07-26

**Authors:** Ibrahim Kaya, Anna Nilsson, Dominika Luptáková, Yachao He, Theodosia Vallianatou, Patrik Bjärterot, Per Svenningsson, Erwan Bezard, Per E. Andrén

**Affiliations:** 1 Department of Pharmaceutical Biosciences, Spatial Mass Spectrometry, Science for Life Laboratory, Uppsala University, Uppsala, Sweden; 2 Section of Neurology, Department of Clinical Neuroscience, Karolinska Institutet, Stockholm, Sweden; 3 University of Bordeaux, CNRS, IMN, UMR 5293, F-33000 Bordeaux, France

**Keywords:** Parkinson's disease, Parkinson's disease

## Abstract

Metabolism of MPTP (1-methyl-4-phenyl-1,2,3,6-tetrahydropyridine) to the neurotoxin MPP^+^ in the brain causes permanent Parkinson’s disease-like symptoms by destroying dopaminergic neurons in the pars compacta of the substantia nigra in humans and non-human primates. However, the complete molecular pathology underlying MPTP-induced parkinsonism remains poorly understood. We used dual polarity matrix-assisted laser desorption/ionization mass spectrometry imaging to thoroughly image numerous glycerophospholipids and sphingolipids in coronal brain tissue sections of MPTP-lesioned and control non-human primate brains (*Macaca mulatta*). The results revealed specific distributions of several sulfatide lipid molecules based on chain-length, number of double bonds, and importantly, hydroxylation stage. More specifically, certain long-chain hydroxylated sulfatides with polyunsaturated chains in the molecular structure were depleted within motor-related brain regions in the MPTP-lesioned animals, e.g., external and internal segments of globus pallidus and substantia nigra pars reticulata. In contrast, certain long-chain non-hydroxylated sulfatides were found to be elevated within the same brain regions. These findings demonstrate region-specific dysregulation of sulfatide metabolism within the MPTP-lesioned macaque brain. The depletion of long-chain hydroxylated sulfatides in the MPTP-induced pathology indicates oxidative stress and oligodendrocyte/myelin damage within the pathologically relevant brain regions. Hence, the presented findings improve our current understanding of the molecular pathology of MPTP-induced parkinsonism within primate brains, and provide a basis for further research regarding the role of dysregulated sulfatide metabolism in PD.

## Introduction

Parkinson’s disease (PD) is a progressive neurodegenerative disease and the most common cause of a movement disorder characterized by motor symptoms including rigidity, bradykinesia, and other cardinal motor features^1^. The main neuropathological characteristics of PD are the loss of dopaminergic neurons in the substantia nigra pars compacta and the presence of cytoplasmic inclusions known as Lewy bodies. The underlying molecular pathogenesis involves multiple pathways and mechanisms such as α-synuclein proteostasis, mitochondrial function, oxidative stress, axonal transport, and neuroinflammation^1,2^. However, we still do not have a complete understanding of the molecular mechanisms that control PD pathogenesis.

In recent years, several lipidomics studies have linked lipids to many aspects of PD pathology, including aggregation, accumulation, and the cytotoxicity of α-synuclein^3–5^. Furthermore, genome-wide association studies have provided new connections between aberrant lipid metabolism and lipid-associated gene-related pathways in PD^6^, e.g., mutations in glucocerebrosidase (GBA) that are linked to a higher risk of developing PD and dementia with Lewy bodies (DLB)^7^. Moreover, many lipidomics studies have reported lipid alterations in post-mortem PD patient brains^8–11^ as well as PD mouse model brains^12,13^. However, these studies were performed using brain tissue extracts, which provide limited spatial information about the detected lipid changes. Therefore, clear in situ spatial distribution profiles are essential to further clarifying the role that lipids play in PD pathogenesis.

Among PD animal models, mitochondrial toxin-based 1-methyl-4-phenyl-1,2,3,6-tetrahydropyridine (MPTP) has been widely used in monkeys and mice to mimic PD symptoms and elucidate the molecular mechanisms of PD pathogenesis, respectively^5^. MPTP inhibits mitochondrial complex I, which leads to mitochondrial dysfunction in dopaminergic neurons. The active metabolite, MPP^+^ (1-methyl-4-phenyl pyridinium ion), enters neurons via the dopamine transporter and causes PD-like symptoms in humans, a dynamic which was first identified in the 1980s^14^. MPTP-treated primate models are a robust phenocopy of PD and recapitulate several aspects of the pathology^15^. These models are now considered the gold-standard of Parkinson’s disease research.

We previously investigated the spatial alterations of neurotransmitters and associated metabolites^16^, carboxyl and aldehyde metabolites^17^, along with neuropeptides^18^, in MPTP-lesioned primate brains using matrix-assisted laser desorption/ionization mass spectrometry imaging (MALDI-MSI). MALDI-MSI allows the simultaneous mapping of the relative abundances and spatial distributions of a wide range of lipid species within their native environment^19^. As such, MALDI-MSI has been a useful tool for investigating how lipids contribute to brain disorders. For example, a number of spatial lipidomics studies have employed MALDI-MSI to improve our understanding of the relationship between lipids and the molecular changes associated with Alzheimer’s disease^20,21^, Hunter’s disease^22^, Huntington’s disease^23,24^, Gaucher disease^25^, Tay-Sachs disease^26^, traumatic brain injury^27,28^, and primary brain tumors^29^.

In the present study, we analyzed samples from a biobank of non-human primate brains (*Macaca mulatta*) representing control monkeys and monkeys in which parkinsonism was induced by MPTP administration^30–32^. We utilized MALDI-Fourier-transform ion cyclotron resonance (FTICR)-MSI in dual polarity (negative and positive ionization modes) to image the distributions of several glycerophospholipids and sphingolipids in coronal brain tissue sections. We discovered distinct distributions of hydroxylated and non-hydroxylated sulfatides within several brain regions. Furthermore, region-specific alterations of hydroxylated and non-hydroxylated sulfatides between the control and MPTP-lesioned primate brain tissues were also visualized. The results demonstrate region-specific depletion in long-chain hydroxylated sulfatides and increases in non-hydroxylated long-chain sulfatides; these differences between control and MPTP-lesioned brain tissues can be attributed to MPTP-induced PD pathogenesis, e.g., peroxisomal α-oxidation of sulfatide lipids and disruption of the myelin sheath, within pathologically relevant brain regions.

## Results

### Distributions of glycerophospholipids and sphingolipids within control brain tissue sections

**Fig. 1:**
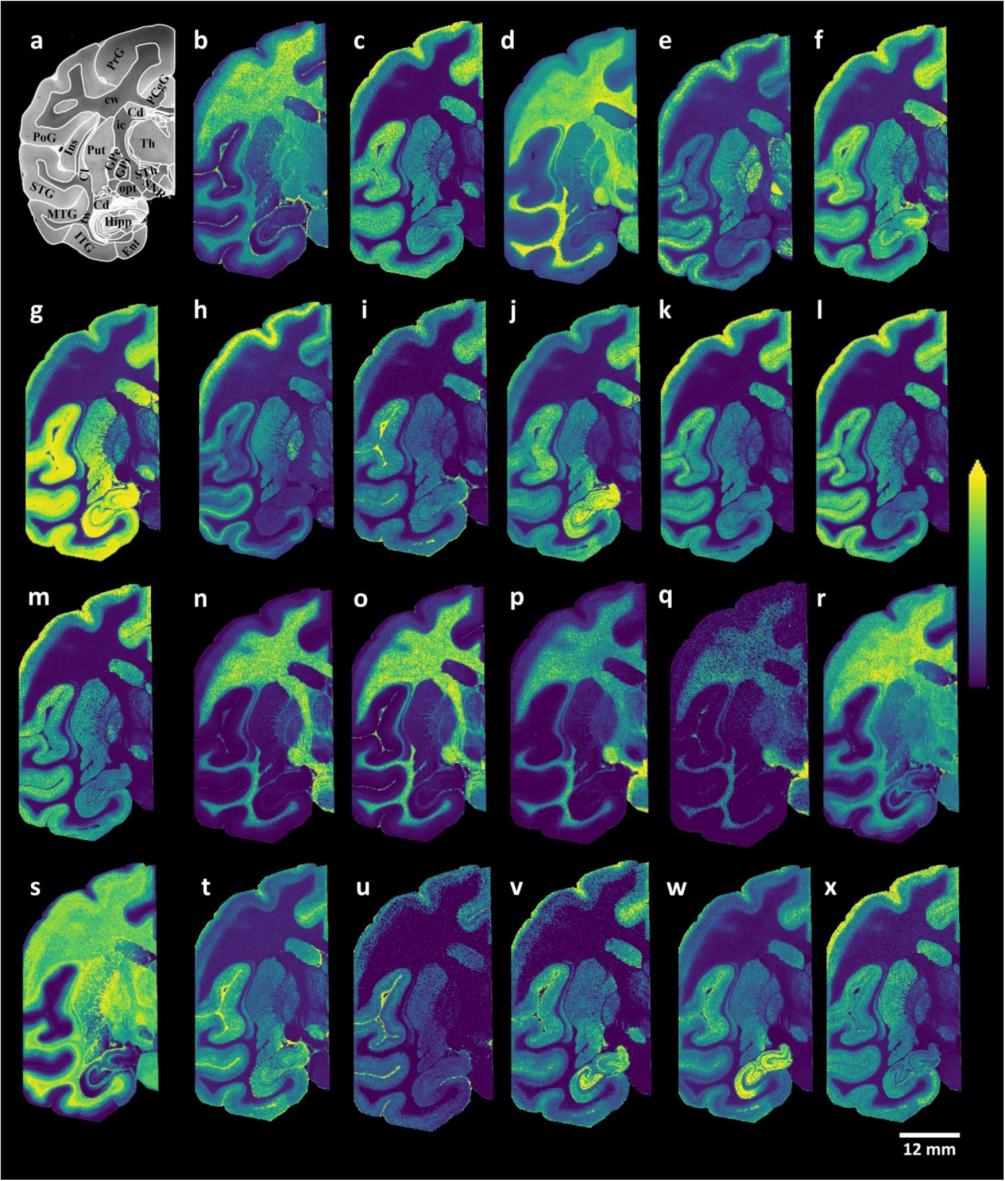
MALDI-MSI images of glycerophospholipid and sphingolipid species. **a** Bright-field image of a macaque brain tissue section with annotated brain regions. Dual polarity MALDI-MSI of the same tissue section (shown in a) reveals the ion images of **b** [PS (36:1)-H]^−^, **c** [PS (40:6)-H]^−^, **d** [PC(36:1) + K]^+^, **e** [PC(40:6) + K]^+^, **f** [PI (36:4)-H]^−^, **g** [PI (38:4)-H]^−^, **h** [PI (40:6)-H]^−^, **i** [PE-NMe_2_ (32:0)-H]^−^, **j** [PE (38:4)-H]^−^, **k** [PE (38:6)-H]^−^]^−^, **l** [PE (40:6)-H]^−^, **m** [PE (P-40:6)-H]^−^, **n** [SM(d42:2) + H]^+^, **o** [SM(d42:1) + K]^+^, **p** [HexCer(d42:2)+Na]^+^, **q** [HexCer(d42:1)+Na]^+^, **r** [SHexCer (d41:1)-H]−, **s** [SHexCer (d43:2)-H]^−^, **t** [CerP (36:1)-H]^−^, **u** [GM3 (36:1)-H]^−^, **v** [GM2 (36:1)-H]^−^, **w** [GM1 (36:1)-H]^−^, **x** [GD1 (36:1)+Na-2H]^−^ in a coronal control macaque brain tissue section. All ion distribution images are scaled to the maximum intensity of each individual ion. PoG postcentral gyrus, PrG precentral gyrus, PCgG posterior cingulate gyrus, STh subthalamic nucleus, STG superior temporal gyrus, MTG middle temporal gyrus, ITG inferior temporal gyrus, Ent entorhinal area, Hipp hippocampus, Cd caudate nucleus, Ins insula, Cl claustrum, Put putamen, GPe/GPi globus pallidus externa/interna, opt optic tract, Th thalamus, ic internal capsule, tw temporal white matter, cw cerebral white matter, SNR substantia nigra pars reticulata. Lateral resolution is 150 µm and all of the ion images were RMS-normalized. The coronal section was obtained at −6 mm relative to the anterior commissure.

**Fig. 2:**
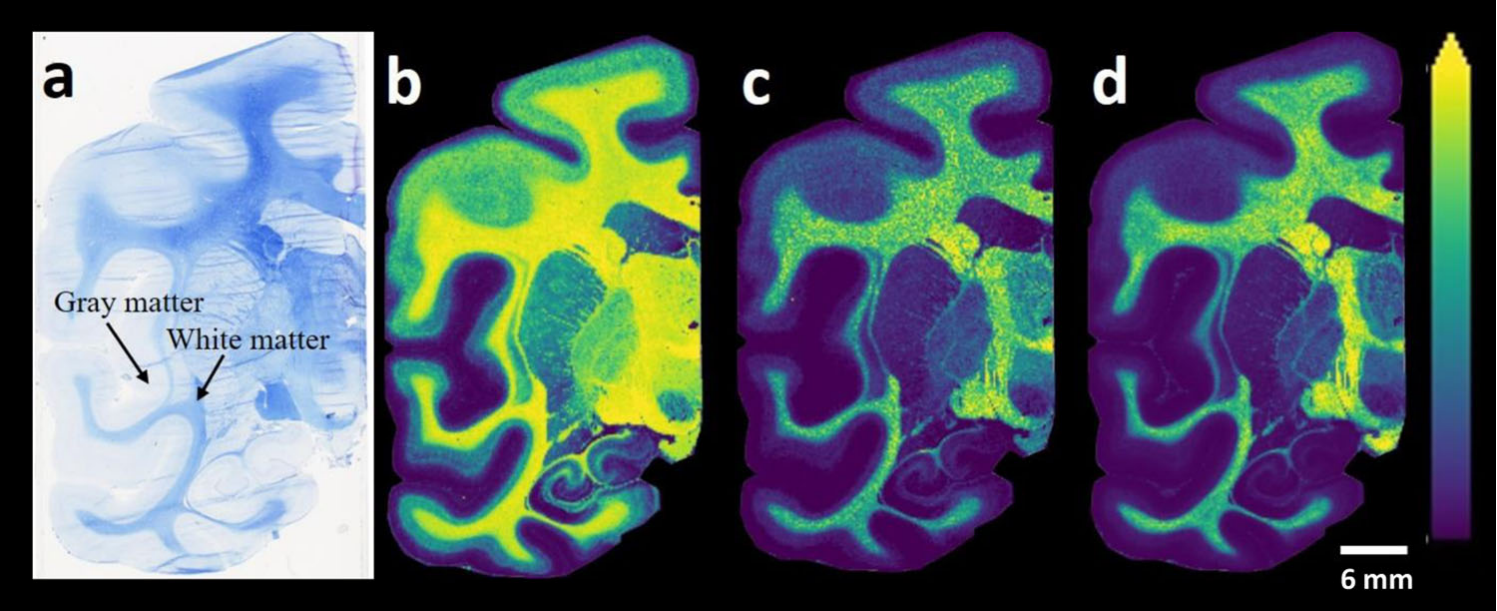
Bright-field stained image and MALDI-MS images of a macaque brain tissue section. **a** Bright-field image of the subsequent luxol fast blue-stained macaque brain tissue section, post-MALDI-MSI analysis. Dual polarity ionization mode MALDI-MSI of the same tissue section reveals the ion distribution images of **b** [SHexCer (d42:2)-H]^−^, **c** [HexCer(d42:2) + K]^+^, and **d** [SM(d42:2) + K]^+^. The ion distribution images are scaled to the maximum intensity of each individual ion. The lateral resolution of the MALDI-MSI images is 150 µm, and all of the ion images were RMS-normalized.

Animal models have provided several fundamental insights into brain aging and neurodegeneration-related brain function alterations^33,34^. The remarkable similarities between humans and macaque monkeys in terms of behavioral capacities, sensory processing abilities, and brain architecture have led to macaque brains becoming the predominant non-human primate model system in brain aging and neurodegeneration research^33,34^. Therefore, it is critical to analyze macaque brain tissue sections with MALDI-MSI to understand the brain region-specific roles of lipids and provide a basis for further research. We initially focused on the lipid species that could be detected and imaged in the control macaque brain tissue sections based on the analysis of both control and MPTP-treated macaque brain tissue sections. The data were obtained from dual polarity MALDI-MSI analyses of coronal sections from one hemisphere of non-human primate brains (150 µm lateral resolution; Fig. 1). Several discrete brain regions, including the postcentral gyrus (PoG), the precentral gyrus (PrG), the posterior cingulate gyrus (PCgG), the subthalamic nucleus (STh), the superior temporal gyrus (STG), the middle temporal gyrus (MTG), the inferior temporal gyrus (ITG), the entorhinal area (Ent), the hippocampus (Hipp), the caudate nucleus (Cd), the insula (Ins), the claustrum (Cl), the putamen (Put), the globus pallidus externa (GPe), the globus pallidus interna (GPi), the optic tract (opt), the thalamus (Th), the internal capsule (ic), the temporal white matter (tw), cerebral white matter (cw), and substantia nigra pars reticulata (SNR) were chosen for the analysis (see Fig. 1a). Several glycerophospholipid species, phosphatidylserine (PS) (Fig. 1b, c), phosphatidylcholines (PC) (Fig. 1d, e), phosphatidylinositol (PI) (Fig. 1f–h), phosphatidylethanolamine (PE) (Fig. 1i–m), and sphingolipid species, includingsphingomyelins (SM) (Fig. 1n, o), hexosylceramides (HexCer) (Fig. 1p, q), sulphated hexosylceramides (SHexCer) (Fig. 1r, s), ceramide phosphates (CerP) (Fig. 1t), monosialogangliosides (GM) (Fig. 1u–w) and disialogangliosides (GD) (Fig. 1x) were visualized within numerous brain regions, including temporal and cerebral cortical areas, along with motor-related brain areas (e.g., the caudate and putamen, the precentral gyrus, the internal and external segments of the globus pallidus, and substantia nigra pars reticulate) (Fig. 1a). Interestingly, PS (36:1) and PC (36:1) were primarily localized to white matter regions (Fig. 1b, d), while longer-chain and polyunsaturated fatty acid-containing PS (40:6) and PC (40:6) species were localized to the grey matter areas (Fig. 1c, f). The PI species PI (36:4), PI (38:4), PI (40:6), PE species PE-NMe_2_ (32:0), PE (38:4), PE (P-40:6), GM and GD species GM3 (36:1), GM2 (36:1), GM1 (36:1), GD1 (36:1), and CerP species CerP (36:1) were predominantly localized within grey matter areas in the coronal macaque brain tissue sections (Fig. 1). As expected, myelin-associated lipid species, including SM species SM(d42:2), SM(d42:1), HexCer species HexCer(d42:2), HexCer(d42:1), and SHexCer species SHexCer (d42:2), SHexCer (d42:1), were localized to the white matter regions (Fig. 1). Subsequent staining of the tissue sections analyzed with dual polarity MALDI-MSI with luxol fast blue (Fig. 2a) revealed that myelin lipid species, including SHexCer (d42:2) (Fig. 2b), HexCer(d42:2) (Fig. 2c), and SM(d42:2) (Fig. 2d), were primarily distributed to myelin-rich brain areas, including the cerebral and temporal white matter areas, the thalamus, the optical tract, and the internal capsule, within coronal macaque brain tissue sections.

### Hydroxylated and non-hydroxylated hexosylceramides displayed distinct distributions within control brain tissue sections

**Fig. 3:**
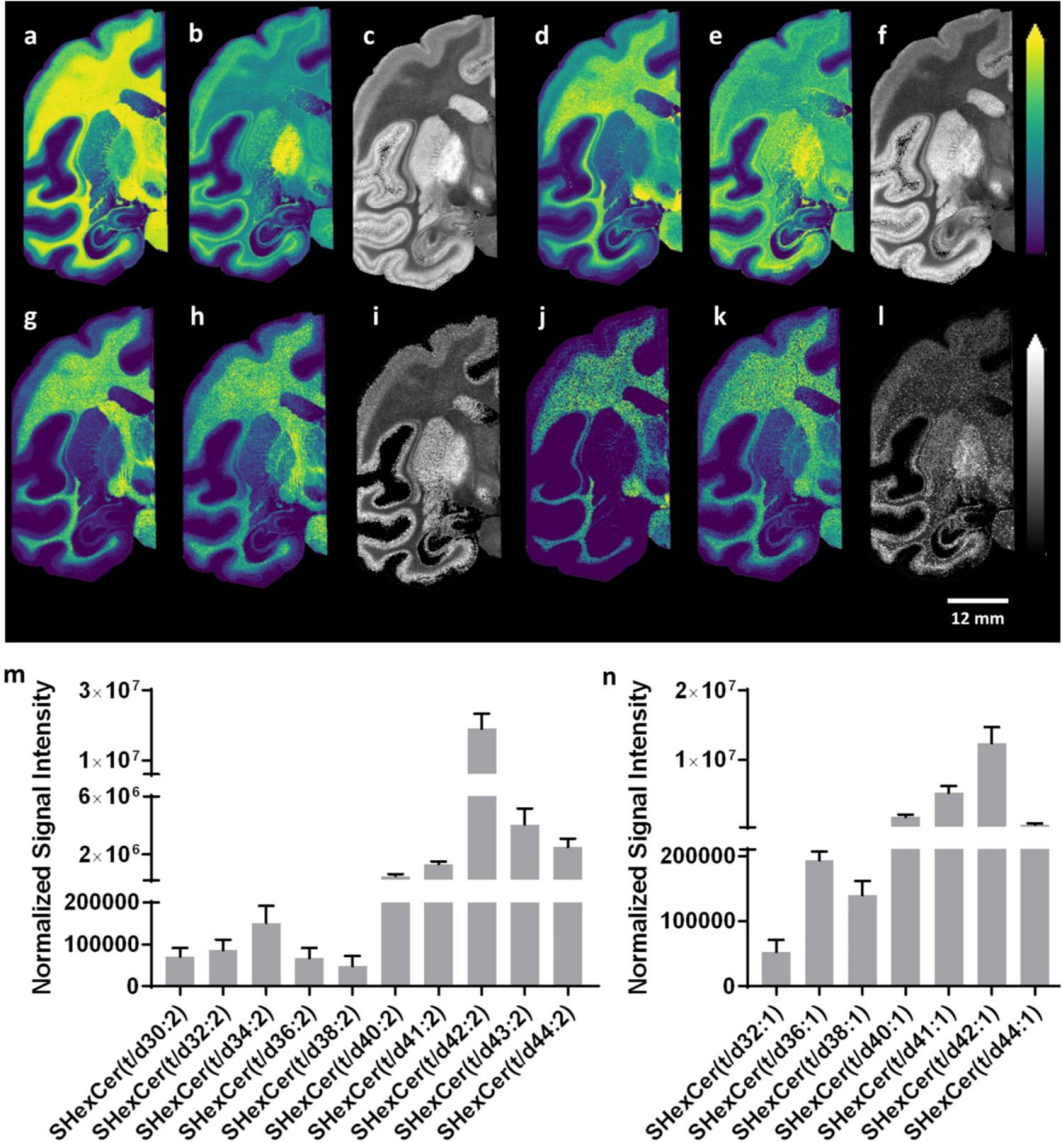
Distributions of hydroxylated and non-hydroxylated sulfatides and hexosylceramides in macaque brain tissue sections. Dual polarity ionization mode MALDI-MSI of the same tissue sections reveals the ion images of **a** [SHexCer (d42:2)-H]^−^, **b** [SHexCer (t42:2)-H]^−^, **c** [SHexCer (t42:2)-H]^−^ normalized to [SHexCer (d42:2)-H]^−^, **d** [SHexCer (d42:1)-H]^−^, **e** [SHexCer (t42:1)-H]^−^, **f** [SHexCer (t42:1)-H]^−^ normalized to [SHexCer (d42:1)-H]^−^, **g** [HexCer (d42:2) + K]^+^, **h** [HexCer (t42:2) + K]^+^, **i** [HexCer (t42:2) + K]^+^ normalized to [HexCer (d42:2) + K]^+^, **j** [HexCer (d42:1) + K]^+^, and **k** [HexCer (t42:1) + K]^+^]. **l** [HexCer (t42:1) + K]^+^ normalized to [HexCer (d42:1) + K]^+^ from macaque brain tissue section at −6 mm relative to the anterior commissure (Fig. 1a). All of the ion distribution images are scaled to the maximum intensity of each individual ion. The lateral resolution of the MALDI-MSI images is 150 µm, and the ion images in (**a**, **b**, **d**, **e**, **g**, **h**, **j**, **k**) were RMS-normalized. Bar graphs (*n* = 5) indicate the signal intensities of individual hydroxylated sulfatides normalized to their non-hydroxylated forms with **m** two and **n** one double bond in the molecular structures in the GPe brain region.

We discovered distinct distributions of hydroxylated and non-hydroxylated sulfatides in the coronal macaque brain tissue sections. Non-hydroxylated sulfatides were predominantly distributed in the white matter areas, while hydroxylated sulfatides were predominantly localized to grey matter areas (Fig. 3). Ion images of hydroxylated SHexCer (t42:2) and SHexCer (t42:1), normalized to non-hydroxylated SHexCer (d42:2) and SHexCer (d42:1), respectively, revealed a relatively high degree of hydroxylation among sulfatides in temporal cortical areas, including ITG, MTG, Ent, STG, as well as motor-related brain areas, including Cd, Put, PrG, GPi, GPe, and SNR (Fig. 3c, f). In line with this, we detected and imaged multiple hydroxylated and non-hydroxylated sulfatides with different chain lengths and numbers of double bonds; the results revealed similar brain tissue distributions for hydroxylated and non-hydroxylated sulfatides, except for SHexCer(36:2) and SHexCer(38:2), for which both non-hydroxylated and hydroxylated forms were abundant in the GP region (Supplementary Fig. 1). Merged images of luxol fast blue-stained macaque brain tissue sections (after MALDI-MSI) and ion images of hydroxylated and non-hydroxylated sulfatides revealed distinct distributions within the white and grey matter areas (Supplementary Fig. 2). Similar distributions of the detected hydroxylated and non-hydroxylated hexosylceramides and sulfatides with the same chain length were observed (Fig. 3g–l). Furthermore, normalizing the signal intensities of hydroxylated sulfatides to their non-hydroxylated forms revealed that hydroxylated forms were more abundant as long-chain sulfatides than shorter-chain sulfatides in motor-related brain regions, such as the GPe (Fig. 3m, n). Highly similar results were observed in some other grey matter regions, including the GPi and SNR brain regions (Supplementary Fig. 3).

### Region-specific alterations of sulfatides in an experimental MPTP-lesioned PD model

To reveal possible region-specific alterations of lipids associated with the experimental PD model, we compared the data, obtained via dual polarity MALDI-FTICR MSI, of control non-human primate brain tissue sections with those of MPTP-treated non-human brain sections^30–32^. Several discrete regions were annotated, e.g., GPi, GPe, Put, Cd, PrG, STh and SNR, based on the involvement of these areas in movement processing; myelin-rich brain areas, e.g., cw, tw, Th, ic and cortical regions, including ITG, MTG, Ent, STG, PrG, PoG, and PCgG, were also annotated. An untargeted mass list including 5225 *m/z* values was obtained from the dual polarity MALDI-FTICR MSI data, and the corresponding average intensities were used to perform a multivariate analysis of GPi, GPe and SNR region, in which the control and MPTP groups were compared. A supervised multivariate modeling approach using partial least squares discriminant analysis (PLS-DA) was applied to highlight differences between the MPTP-treated and control brain sections (Supplementary Fig. 4). Following optimization of the PLS-DA models, *m/z* values with high VIP values were selected for identification (Supplementary Table 1). The detected *m/z* values with high VIP values were initially identified by accurate mass matching against databases, with the identification confirmed by on-tissue MS/MS. Several m/z values heavily contributed to the separation of the control and MPTP groups; interestingly, majority of these values were particularly identified as long-chain (≥40) hydroxylated or non-hydroxylated sulfatides. Therefore, we focused our subsequent statistical investigation on region-specific changes of hydroxylated and non-hydroxylated sulfatides.

**Fig. 4:**
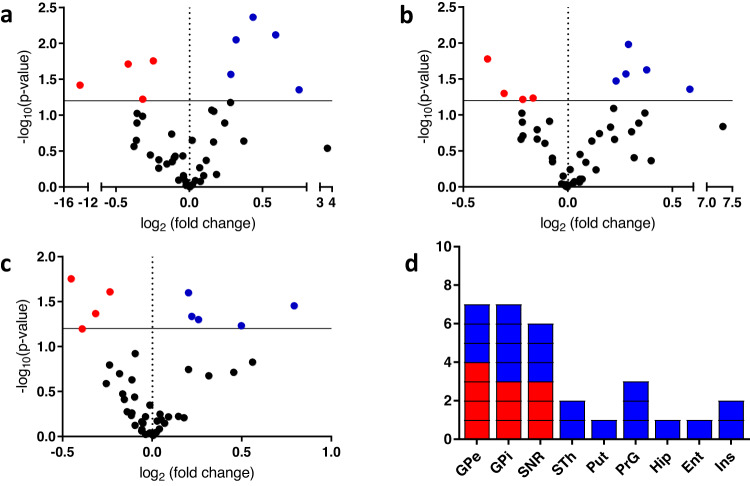
Targeted statistical data analysis of hydroxylated and non-hydroxylated sulfatides. A targeted lipidomics workflow for sulfatides was designed to identify hydroxlated and non-hydroxylated sulfatides that differed between the control and MPTP groups. **a–c** Volcano plots produced using all of the identified hydroxylated and non-hydroxylated sulfatides (Supplementary Table 1) to evaluate possible significant between-group differences of hydroxylated (red) and non-hydroxylated (blue) sulfatides in **a** GPi, **b** GPe, and **c** SNR. **d** Stacked bars indicate nine brain regions which exhibits significant (*P* ≤ 0.05) or near statistically significant (very close values to *P* = 0.05) as evaluated as significant using multiple *t* tests between-group (MPTP-lesioned and control brain) changes (Supplementary Table 2) in long-chain (≥40) hydroxylated (red) and long-chain (≥40) non-hydroxylated (blue) sulfatides. Each long-chain (≥40) sulfatide which has either red (hydroxylated) or blue (non-hydroxylated) may appear multiple times if its abundance differed significantly between groups in more than one region.

**Fig. 5:**
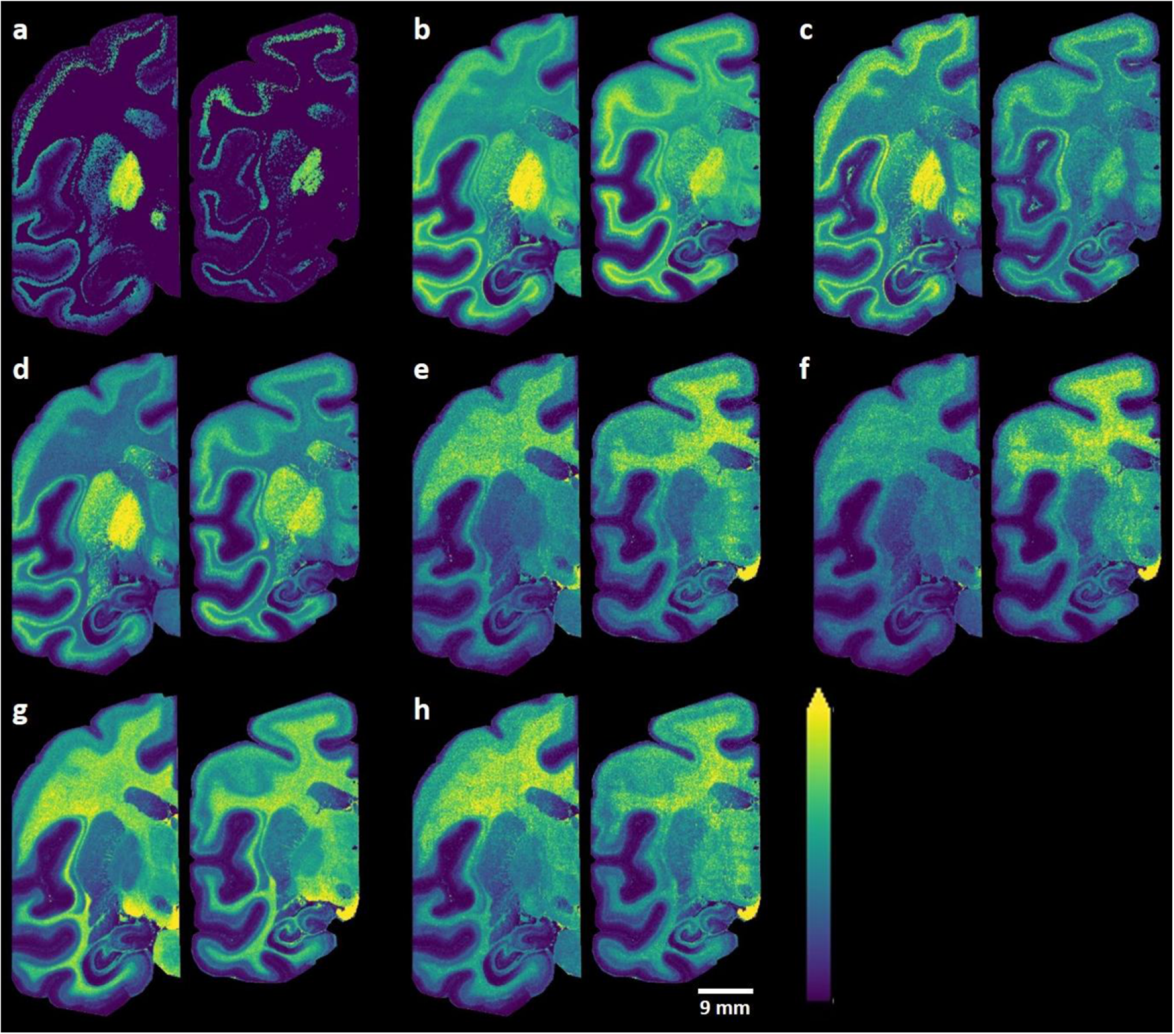
MALDI-MS ion distribution images of sulfatides that showed significant differences between control (left) and MPTP-lesioned (right) brain sections. **a** [SHexCer (t41:2)-H]^−^, **b** [SHexCer (t42:2)-H]^−^, **c** [SHexCer (t42:3)-H]^−^, **d** [SHexCer (t43:2)-H]^−^, **e** [SHexCer (d40:1)-H]^−^, **f** [SHexCer (d40:2)-H]^−^, **g** [SHexCer (d42:1)-H]^−^, **h** [SHexCer (d41:1)-H]^−^. Ion distribution images are scaled to the maximum intensity of each individual ion. Lateral resolution of the MALDI-MSI images is 150 µm and all of the ion images were RMS-normalized.

Long-chain hydroxylated and non-hydroxylated sulfatides within the GPe, GPi, and SNR regions were among the sulfatides that showed the largest differences between MPTP-treated and control brain sections (Fig. 4 and Supplementary Table 1). More specifically, the MPTP group showed a lower abundance of certain long-chain hydroxylated sulfatides with polyunsaturated chains, including SHexCer (t41:2), SHexCer (t42:2), SHexCer (t42:3), and SHexCer (t43:2), in the GPi, GPe, and SNR regions relative to the control group (Fig. 5a–d). In contrast, certain long-chain non-hydroxylated sulfatides, including SHexCer (d40:1), SHexCer (d40:2), SHexCer (d42:1), and SHexCer (d41:1), were present at higher levels in multiple brain regions, e.g., GPi, GPe, and SNR, in the MPTP group when compared to the control group (Fig. 5e–h and Supplementary Table 2). The distributions of several sphingolipids and glycerophospholipids that did not display significant changes between the control and MPTP groups were also recorded (Supplementary Fig. 5).

## Discussion

This study applied the MALDI-MSI technique to a comprehensive lipidomics analysis of coronal macaque brain tissue sections representing the dynamics of PD. We report distinct distributions of several hydroxylated and non-hydroxylated sulfatides with different acyl chain lengths within several brain regions. A significant finding was that hydroxylated sulfatides were found to show predominant distribution within grey matter areas, particularly within motor-related brain regions and temporal cortical areas. Furthermore, the abundance of hydroxylated sulfatides increased at the border between white and grey matter. Distinct distributions of hydroxylated and non-hydroxylated sulfatides in the white and grey matter have previously been described in experiments of human cerebral cortex tissue sections^35^. Our findings enhance the understanding of lipid distribution in the brain by providing a comprehensive mapping of hydroxylated and non-hydroxylated sulfatides, not only on the borders of the grey and white matter, but also among distinct brain regions in coronal macaque brain tissue sections.

Following the discovery of distinct distributions of hydroxylated and non-hydroxylated sulfatides, we noticed the depletion of certain long-chain hydroxylated sulfatides in specific brain regions, including GPe, GPi, and SNR. In contrast, certain non-hydroxylated sulfatides were present at elevated levels in multiple brain regions, including, GPe, GPi, and SNR, within the MPTP-treated primate brain tissue when compared to control sections. Previous lipidomics studies of post-mortem PD patients have reported elevated levels of sulfatides in the superior frontal and cerebellar grey matter^36^, while a recent study reported a reduction in sulfatides in the lipid rafts of human grey matter in incidental PD (with Lewy body pathology in the brain stem without motor symptoms)^8^. However, these studies did not include descriptions of whether these changes in sulfatides showed a discernible trend with chain length, number of double bonds, or hydroxylation stage; spatial information about the changes across multiple brain regions was also limited. Prior studies have also reported significant depletion of sulfatides in Alzheimer’s disease (AD), without descriptions of the link to chain length, number of double bonds, or hydroxylation stage^37,38^; moreover, there is strong evidence that these changes in sulfatides in the AD brain are associated with apolipoprotein E (APOE)-related mechanisms within amyloid plaques^21,39^.

MPTP is a protoxin that crosses the blood–brain barrier and is converted to MPP^+^ via the action of monoamine oxidase B (MAO-B); MPP+ then enters dopaminergic neurons and leads to apoptosis^40^. While it has been widely reported that the loss of these neurons in PD is associated with a glial response that mainly comprises activated microglial cells and reactive astrocytes^41^, only a few reports suggest that oligodendrocytes are involved in PD^41^. Oligodendrocytes in the striatum are damaged by MPTP neurotoxicity in mice^42^. This indicates that MPTP-induced pathology can be also detrimental to oligodendrocytes within the grey matter. Therefore, the depletion of long-chain hydroxylated sulfatides within motor-related grey matter regions (GPe, GPi, and SNR) in the MPTP-lesioned macaque brain might be associated with the disruption of the myelin sheath around the axons of neurons. It is known that peroxisomal α-oxidation can be involved in the degradation pathway of hydroxyl fatty acids within sphingolipids, including hydroxylated sulfatides, and an enzyme in this process can catalyze the one-carbon cleavage of hydroxylated fatty acid in the peroxisome, which would result in non-hydroxylated fatty acids that can be reused during sphingolipid synthesis in the endoplasmic reticulum^43^. These processes can alter the composition of hydroxylated and non-hydroxylated sulfatides within the brain^44^. Furthermore, this oxidation process in peroxisomes is specific to long-chain fatty acids^45^, and lipids containing polyunsaturated fatty acids are more susceptible to oxidation during neurodegeneration^46,47^. Previous research has provided strong evidence that peroxisome dysfunction is associated with the lipid metabolism dysregulation that occurs in PD and some other neurodegenerative diseases^48,49^. Therefore, the changes in long-chain hydroxylated and non-hydroxylated sulfatides observed in the MPTP-lesioned macaque brain could be the result of MPTP-induced peroxisomal α-oxidation within the GPe, GPi, and SNR. Nevertheless, it has also been reported that sulfatides can stimulate inflammatory mechanisms within immune cells, such as microglia and astrocytes^50^. Therefore, it is also possible that shifts in the levels of certain sulfatides, a result of dysregulated metabolism, within pathological regions of the MPTP-lesioned macaque brain exacerbated inflammation-related pathological conditions.

It was previously reported that short-chain sulfatides (total carbon chain length of sphingosine base and fatty acid <40), mainly hydroxylated and non-hydroxylated SHexCer (36:1), dominate in astrocytes, neurons, and pro-oligodendroblasts^51,52^, while long-chain (≥40) sulfatides are more common in mature oligodendrocytes^52^. We observed a higher abundance of hydroxylated short-chain sulfatides in the grey matter areas in which astrocytes and neurons are abundant, whereas non-hydroxylated short-chain sulfatides were strongly distributed in the white matter areas where mature oligodendrocytes and myelin are abundant^53^. The distributions of double-bond containing hydroxylated and non-hydroxylated SHexCer(36:2) and SHexCer(38:2) revealed that these species are predominantly localized to the GP region in grey matter; this indicates that the distribution of sulfatides within macaque brain tissue depends on specific chain length and the number of double bonds. We also observed a relatively high abundances of certain non-hydroxylated long-chain sulfatides with multiple double bonds, including SHexCer (d42:3), SHexCer (d43:2), SHexCer (d44:2), and SHexCer (d44:3), at the border between white and grey matter.

Within the central nervous system (CNS), sulfatides are the predominant sulfoglycophingolipids in the myelin sheath^54^; as such, the abundance of sulfatides is proportional to the amount of myelin present in the rat brain^55^. The examination of mice deficient in cerebroside sulfotransferase (CST), a sulfatide-synthesizing enzyme, provided novel insight into the critical role of sulfatides in the differentiation of myelinating cells, formation of the paranodal junction, nerve conduction, myelin maintenance, and myelin lipid synthesis by oligodendrocytes^56–59^. Although widely regarded as a marker for oligodendrocytes in the CNS and Schwann cells in the peripheral nervous system, sulfatides are also present at relatively small amounts in astrocytes and neurons^60^. Sulfatides demonstrate a certain degree of structural variety, with the most common features varying acyl chain length and differences in the ceramide moiety, which can be hydroxylated^61^. These subtle structural differences are important for precise CNS function^61^. Hydroxylated sulfatides are prevalent in the nervous system^62^, and the hydroxylation of sulfatide N-acyl chains, which is catalyzed by fatty acid 2-hydroxylase (FA2H), occurs during de novo ceramide synthesis^63^. Mutations in the *FA2H* gene were found to be associated with neurodegeneration, such as leukodystrophy with spastic paraparesis, and dystonia^64^. This suggests that the hydroxylation state of sulfatides plays an important role in CNS function, and hence, neurodegeneration; therefore, knowledge of the brain region-specific distributions of both hydroxylated and non-hydroxylated sulfatides would be important prior to clinical studies to understand specific biological functions.

Our data clearly show that the distribution of sulfatides varies depending on hydroxylation stage; for instance, hydroxylated sulfatides demonstrated predominant localization to grey matter regions relative to white matter brain regions. This suggests that the mechanisms of sulfatide hydroxylation in oligodendrocytes differ between grey and white matter regions^35^. In line with this, studies using neonatal rodent brains revealed that the ratio of hydroxylated/non-hydroxylated sulfatides increases during myelination^65,66^. Oligodendrocytes lie in longitudinal arrays in the white matter, whereas grey matter oligodendrocytes frequently demonstrate a satellite formation around neurons, wrap around nerve axons, and possibly help regulate the neuronal microenvironment^53,67^. Our data show that long-chain hydroxylated sulfatides must be associated with myelination in the grey matter, particularly in the motor-related brain regions.

To summarize, we imaged several intact glycerophospholipids and sphingolipids using dual polarity ionization mode MALDI-MSI and reported the associated distributions within several brain regions in coronal brain tissue sections from non-human primate brains (control and MPTP-lesioned). The results revealed specific distributions of several sulfatide lipid molecules based on chain length, number of double bonds, and hydroxylation stage. Certain long-chain hydroxylated sulfatides with polyunsaturated chains in the molecular structure were specifically depleted within certain motor-related brain regions (GPe, GPi, and SNR), while certain long-chain non-hydroxylated sulfatides were found at elevated levels within several brain regions (GPe, GPi, and SNR). This revealed region-specific dysregulation of sulfatide metabolism within the MPTP-lesioned macaque brain. The depletion of long-chain hydroxylated sulfatides with polyunsaturated chains in the molecular structure could be explained by MPTP-induced peroxisomal oxidation within pathologically relevant brain regions (e.g., GPe, GPi, and SNR). The dysregulation of sulfatides can perturbate the myelin sheath around the axons of the neurons, which can be expected to negatively affect neural conduction. The presented results have improved our overall understanding of the MPTP-induced molecular pathology of parkinsonism within primate brains, and provides a strong basis for further research into how dysregulated sulfatide metabolism influences PD development and progression.

## Methods

### Chemicals and reagents

All of the chemicals used in sample preparation were of pro-analysis grade and obtained from Sigma-Aldrich (St. Louis, MO) unless otherwise specified.

### Animal experiments

All of the animal experiments were carried out following the European Communities Council directive of November 24, 1986 (86/609/EEC), revised in 2010 (2010/63/EU), for laboratory animal care in an AAALAC-accredited facility after acceptance of the study design by the Institute of Lab Animal Science (IACUC; Chinese Academy of Science, Beijing, China) for experiments conducted on non-human primates. An experienced and skilled veterinarian supervised the animals’ care and health at all times.

The non-human primate brain tissue samples analyzed in this study were obtained from a previously published biobank of female rhesus monkeys (*Macaca mulatta*, Xierxin, Beijing, PR of China) with an age of 5 ± 1 years and mean weight of 5.3 ± 0.8 kg^30–32^. Parkinsonism was induced according to published protocols^68,69^; more specifically, the animals received daily MPTP injections, 0.2 mg/kg intravenously (i.v.), until stable parkinsonism was established. Control animals (*n* = 5) received daily saline injections instead of MPTP. Parkinsonism symptoms were evaluated using a clinical rating scale optimized for macaques^70^. Animals in the group referred to as MPTP (*n* = 5) received no further treatment after PD symptoms stabilized. All of the animals were euthanized six months after the first MPTP exposure. Brains were quickly removed and frozen in dry ice-cooled isopentane (−45 °C), and care was taken to ensure that the time between euthanasia and sample freezing was 10 min for all the animals. The two hemispheres were separated, with one stored at −80 °C until sectioning for MALDI-MSI analysis.

### Tissue processing

For MALDI-MSI, coronal brain tissue sections (−6 mm from the anterior commissure)^71^ from one hemisphere of all control and MPTP-treated animals were sectioned at a thickness of 12 μm in a cryostat (Leica CM3050S, Leica Microsystems, Wetzlar, Germany) at −20 °C. Sections were then thaw-mounted onto indium tin oxide-coated glass slides (Bruker Daltonics, Bremen, Germany) for MALDI-MSI and stored at −80 °C prior to analysis.

### Design of MALDI-MSI experiments

Each control sample was analyzed together with an MPTP sample on direct consecutive days under the same experimental conditions. All pairs were randomly selected, and the order in which the control and MPTP samples were analyzed was random. We analyzed coronal brain sections taken at −6 mm relative to the anterior commissure^71^; this enabled the investigation of motor-related brain areas, including Cd, Put, PrG (where the primary motor cortex is located), GPi and GPe), along with STh and SNR. Ion images of some lipid species from all control (*n* = 5) and MPTP-treated macaque brain (*n* = 5) were displayed in Supplementary Fig. 6.

### Sample preparation for MALDI-MSI

Sections were desiccated at room temperature for 15 min before the spray coating of a norharmane matrix solution for dual polarity analysis of lipids^72^. Prior to matrix coating, the slide was scanned on a flatbed scanner (Epson Perfection V500, Nagano, Japan). The matrix solutions were prepared by dissolving the norharmane matrix powder in 80% MeOH (7.5 mg/ml) solution in a glass vial, followed by brief sonication. An automated pneumatic sprayer (TM-Sprayer, HTX-Technologies LLC, Chapel Hill, NC, USA) was used, and combined with a pump (AKTA FPLC P-905, Amersham Pharmacia Biotech, Uppsala, Sweden) to spray the heated matrix solution over the tissue sections. The pump was kept running at 70 μL/min using a 50% ACN pushing solvent with isocratic pressure before the experiments to ensure a stable flow of the solvent. The matrix solution was sprayed using the following instrumental parameters: solvent flow rate of 70 μL/min; nitrogen pressure of 6 psi; nozzle spray temperature of 60 °C; 15 passes (all horizontal); nozzle head velocity of 1200 mm/min; and track spacing of 2.0 mm.

### MALDI-MSI analysis

All of the MALDI-MSI experiments for lipid imaging were performed in both negative and positive ionization modes on the same tissue sections using a MALDI-FTICR (7T solariX XR-2ω, Bruker Daltonics) mass spectrometer equipped with a Smartbeam II 2 kHz laser. The size of the laser was chosen to give a lateral resolution of 150 μm in both polarities, with an offset value of 75 μm to ensure no laser ablation overlaps when switching between polarities. The instrument was tuned for optimal detection of lipid molecules (*m/z* 200–2000) in both polarities using the quadrature phase detection (QPD) (2ω) mode. The transient length was 0.73 s, providing a mass resolution of about 106000 at *m/z* 850 during the MSI analysis of lipids on the brain tissue sections.

The time-of-flight values were set at 0.8 ms and 1.0 ms for positive and negative ion mode analysis, respectively, and the transfer optics frequency was kept at 4 MHz for both polarities. The quadrupole isolation *m/z* value (Q1 mass) was set at *m/z* 220.00 for both polarity modes. In both polarity modes, spectra were collected by summing 100 laser shots per pixel. Both methods were calibrated externally with red phosphorus over an appropriate mass range. Ion signals of *m/z* 885.549853 (monoisotopic peak of [PI(38:4)-H]^−^) and *m/z* 798.540963 (monoisotopic peak of [PC(34:1) + H]^+^) were used for the internal calibration of negative and positive polarity analyses, respectively. The laser power was optimized at the start of each analysis and then held constant during the MALDI-MSI experiment.

### Tissue staining

A Luxol fast blue staining kit (Abcam, ab150675; Abcam, Cambridge, UK) was used with a similar protocol as previously described for myelin staining^73^. Briefly, after MALDI-FTICR-MSI, the slides were immersed in methanol for about 1 min to remove remnants of the norharmane matrix from the tissue sections. Then, the slides were immersed in the Luxol fast blue solution for 2 h at 60 °C, rinsed and differentiated according to the staining protocol, and incubated in Cresyl Echt violet for 5 min to visualize neurons. The slides were then rapidly dehydrated in absolute alcohol, cleared, and mounted with DPX (Merck, Darmstadt, Germany). The sections were scanned using a high-resolution slide scanner (Epson Perfection V750 PRO).

### Data processing and analysis

MALDI-MSI data from five pairs of control and MPTP-lesioned samples were visualized in FlexImaging (v. 5.0, Bruker Daltonics). For further analysis, data were imported to SCiLS Lab (v. 2020a Pro, Bruker Daltonics), in which brain regions were annotated according to a stereotaxic template atlas of the macaque brain^71^. We utilized the sliding window function of SCiLS Lab to extract root mean square (RMS) normalized average peak area values for 2520 peaks in the negative ion mode and 2705 peaks in positive ion mode separately. The peaks lists were combined into an excel file for multivariate data analysis. To obtain an initial understanding of the data, principal component analysis (PCA) was conducted (SIMCA 15.0, Sartorius Stedim Biotech, Umeå, Sweden) after applying scaling (pareto scaling) and log transforming the data^74^. The Hotelling T2 ellipse (T2Crit) and distance to model (DModX; with a 95% confidence interval) were used as criteria for outlier detection. Subsequently, PLS-DA was applied to identify MPTP-induced brain lipid alterations in the regions of GPi, GPe and SNR (Supplementary Fig. 4). The variable selection was based on the variable influence to projection (VIP > 1), the weight (w) in the loadings plot, and the size of coefficients. Robust models should demonstrate a difference between R2 and the cross-validated R2, i.e., Q2, that is lower than 0.2. The PLS-DA models underwent validation using cross-validation ANOVA, with a significance level *P* < 0.05 indicating a robust model. Additionally, permutation tests were performed (100 permutations). Univariate analysis, i.e., two-tailed *t* test on log10 transformed data, was performed on the lipids indicated significant by the PLS-DA. All other statistical analyses were performed using GraphPad Prism (GraphPad Software, La Jolla, California, USA, v. 8.4). Before hypothesis testing, the data normality was checked using the Shapiro Wilk normality test. Multiple *t* tests were used to evaluate the significance of between-group differences in the abundance of identified sulfatides. Log2 fold changes (FC) were calculated based on the ratio of the average abundance of a sulfatide in the MPTP sections to the average abundance in the control sections.

### Image co-registration

Bright-field luxol fast blue-stained images and MALDI-MSI ion images of single coronal macaque brain tissue sections were overlaid using fiducial markers using the co-register image function of FlexImaging (v. 5.0 (Bruker Daltonics).

### Identification of lipids

An untargeted mass list including 5225 *m/z* values was used as an input to the PLS-DA analysis. The *m/z* values with high VIP values (Supplementary Table 1) were selected for identification and searched against the LIPID MAPS database with a 0.01 m/z tolerance for both negative and positive polarities, including all ion types. Through mass accuracy and MS/MS analysis, several *m/z* values with high VIP scores (Supplementary Table 1) were identified as SHexCers. Hence, our research focused on SHexCers. However, it should be noted that not all SHexCer species are included in the LIPID MAPS database. Therefore, we also included additional hydroxylated and non-hydroxylated SHexCer species from published literature, which are not present in LIPID MAPS^35,75^. In a separate analysis, we included several identified glycerophospholipids and sphingolipids to examine their distributions in the brain tissue sections. Consequently, they have been included in our lipid assignment list (see Supplementary Table 3). No deisotoping of the spectra was applied prior to lipid assignments. MALDI-tandem MS (MS/MS) was performed on tissues by collecting spectra from brain regions where the target ion was abundant. The resulting product ions were then compared to product ion spectra of standards in the LIPID MAPS database (Nature Lipidomics Gateway, www.lipidmaps.org) and/or previously published data^76,77^. In cases where sodium and/or potassium adduct ions of the same lipid species were identified, the brain tissue distribution of the adducts and [M + H]^+^ ions were compared (Supplementary Figs. 6–16). Lipids were assigned based on mass accuracy in instances where the peak intensity was insufficient for MS/MS analysis of the tissue. The mass error in the corresponding mass range was determined through MS/MS analysis of the nearby *m/z* values of other lipid species, including SHexCers. Fatty acid chain or long-chain base-specific information of some of the lipids were obtained by MS/MS (Supplementary Table 3). For a detailed explanation of lipid annotations refer to Supplementary Tables 3 and 4.

## Supplementary information

Supplementary Information

Data Set

## Data Availability

The source data including the average peak area of the lipids in all animals is available online in the Supplementary Data. The MALDI-MSI datasets used and/or analyzed during the current study available from the corresponding author on request.
