# Supplementary material for: Spatial lipidomics reveals brain region-specific changes of sulfatides in an experimental MPTP Parkinson’s disease primate model
Source: NPJ Parkinsons Dis. 2023 Jul 26;9:118. doi: 10.1038/s41531-023-00558-1 (PMC10372136; doi:10.1038/s41531-023-00558-1)
Supplement: Supplementary file 1 — Supplementary Information [file 41531_2023_558_MOESM1_ESM.pdf]

SUPPLEMENTARY INFORMATION

Spatial lipidomics reveals brain region-specific changes of sulfatides in an experimental MPTP Parkinson's disease primate model

Ibrahim Kaya[1], Anna Nilsson[1], Dominika Luptáková[1], Yachao He[2], Theodosia Vallianatou[1], Patrik Bjärterot[1], Per Svenningsson[2], Erwan Bezard[3], Per E. Andrén[1]*

1. Department of Pharmaceutical Biosciences, Spatial Mass Spectrometry, Science for Life Laboratory, Uppsala University, Uppsala. Sweden
2. Section of Neurology, Department of Clinical Neuroscience, Karolinska Institutet, Stockholm, Sweden.
3. Univ. Bordeaux, CNRS, IMN, UMR 5293, F-33000 Bordeaux, France

Corresponding Author

Per E. Andrén

Department of Pharmaceutical Biosciences, Spatial Mass Spectrometry, Science for Life Laboratory, Uppsala University, Uppsala, Sweden

Email, per.andren@uu.se

Phone, +46-70 167 9334

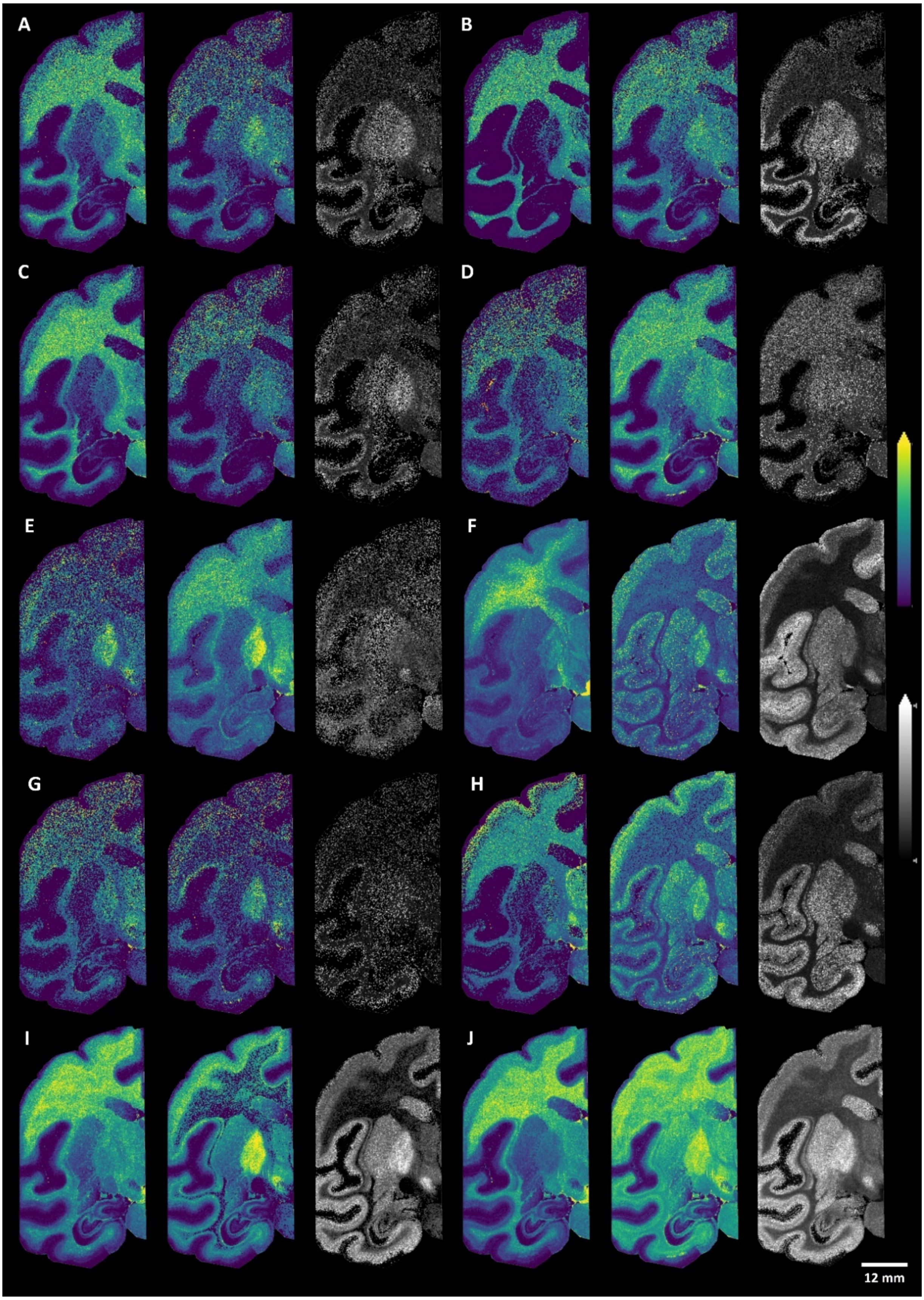
A
B
C
D
E
F
G
H
I
J
12 mm

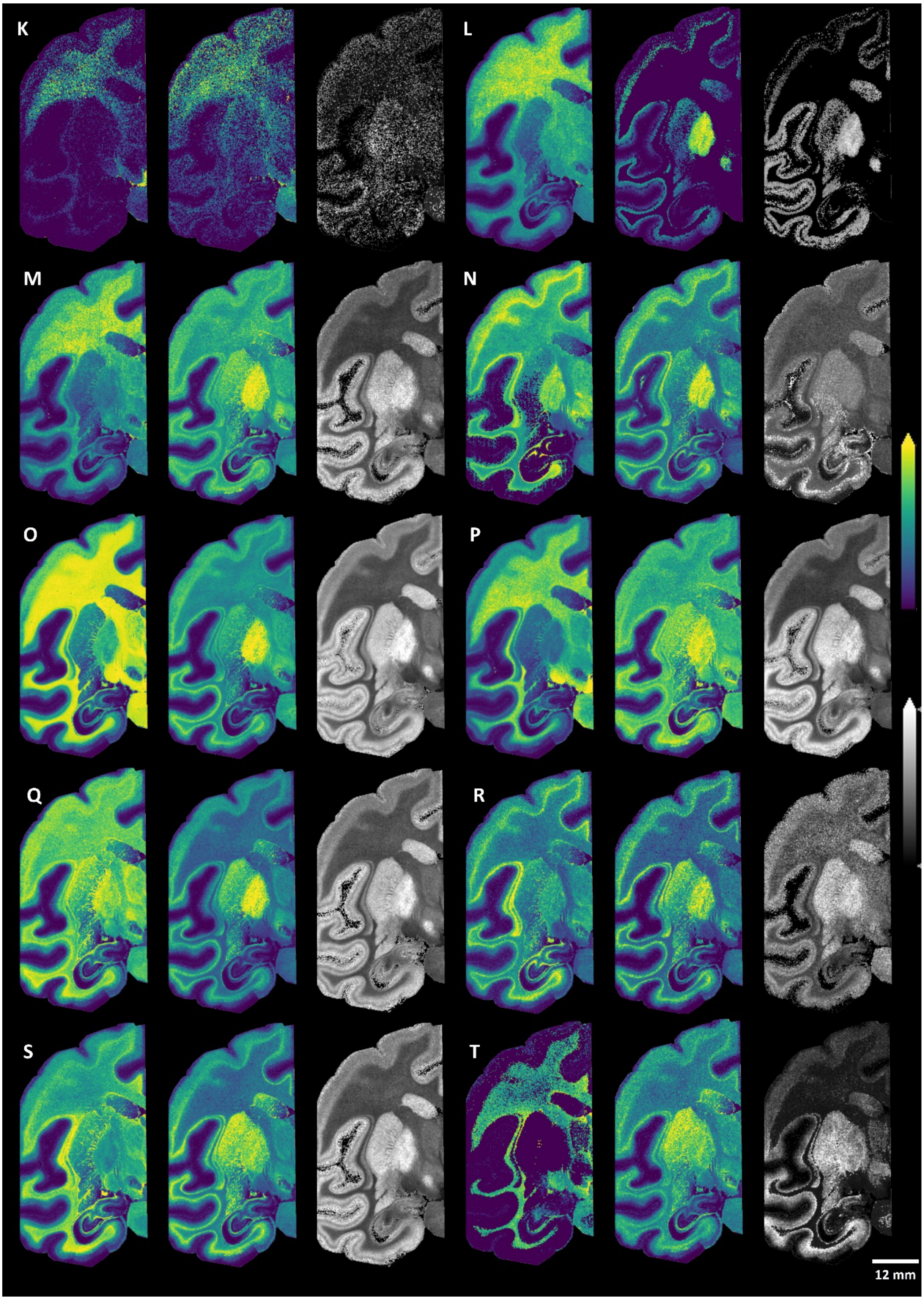
K
L
M
N
O
P
Q
R
S
T
12 mm

Supplementary Figure 1. MALDI-FTICR-MSI provides lateral ion distributions images of non-hydroxylated sulfatides (left). hydroxylated sulfatides (middle) and hydroxylated sulfatides normalized to non-hydroxylated sulfatides (right).

a SHexCer (30:2), **b** SHexCer (32:2), **c** SHexCer (32:1), **d** SHexCer (34:2), **e** SHexCer (36:2), **f** SHexCer (36:1), **g** SHexCer (38:2), **h** SHexCer (38:1), **i** SHexCer (40:2), **j** SHexCer (40:1), **k** SHexCer (40:0), **l** SHexCer (41:2), **m** SHexCer (41:1), **n** SHexCer (42:3), **o** SHexCer (42:2), **p** SHexCer (42:1), **q** SHexCer (43:2), **r** SHexCer (44:3), **s** SHexCer (44:2), and **t** SHexCer (44:1). All images in the left and middle panel were RMS-normalized and scaled to the maximum intensity of each ion. Lateral resolution is 150 μm.

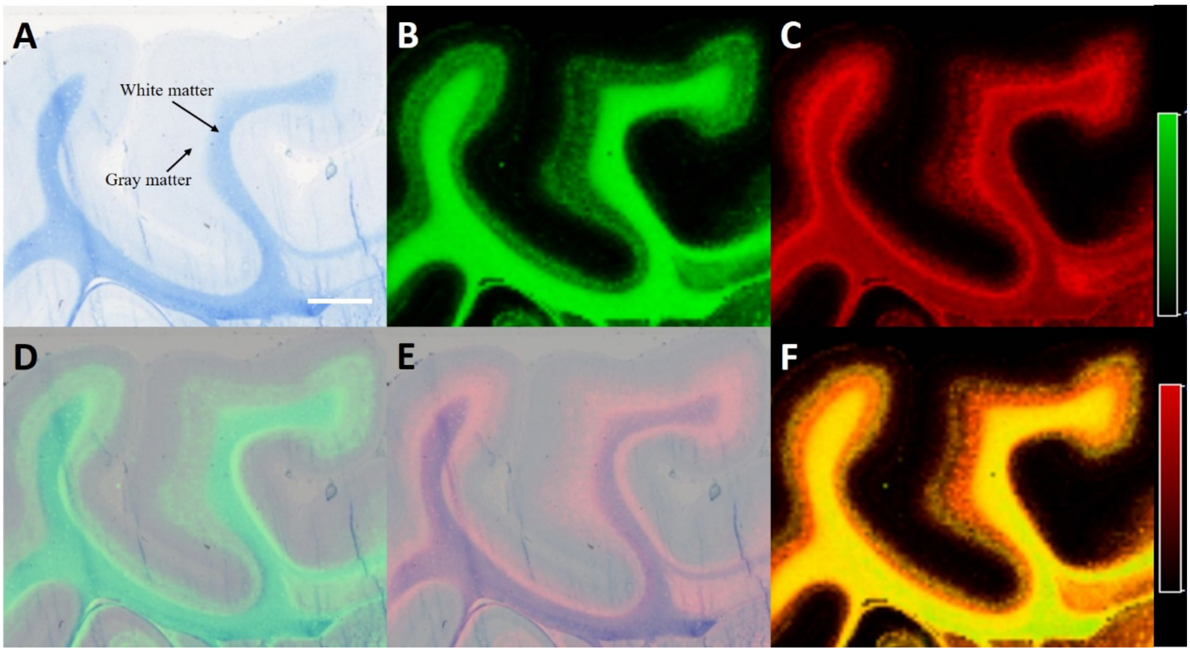

Supplementary Figure 2. Overlaying ion images of non-hydroxylated sulfatides and hydroxylated sulfatides obtained with MALDI-FTICR-MSI and the microscopy images of the luxol fast blue myelin staining of the same coronal macaque tissue sections.

a Luxol fast blue staining of the same tissue section analyzed with MALDI-FTICR-MSI, ion images of **b** SHexCer (d42:2), **c** SHexCer (t42:2), overlaid ion images of **d** SHexCer (d42:2) and **e** SHexCer (t42:2) with the microscopy image of luxol fast blue myelin staining. **f** Merged ion images of SHexCer (d42:2) (green), SHexCer (t42:2) (red). All the ion images were RMS-normalized and scaled to the maximum intensity of each ion. Lateral resolution is 150 µm. Scale bar is 3 mm.

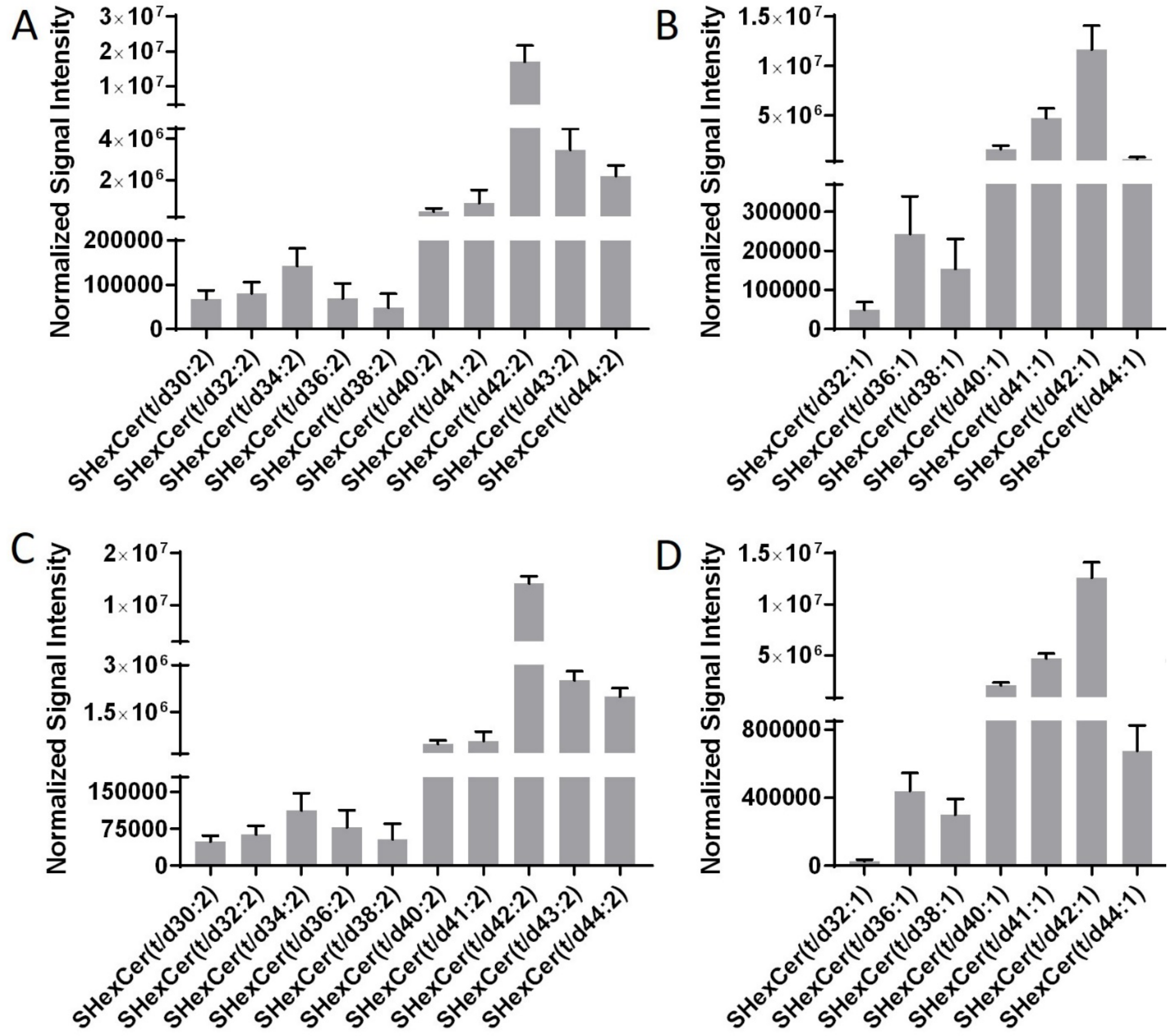

Supplementary Figure 3. Bar graphs (n=5) indicate the signal intensities of individual hydroxylated sulfatides normalized to their non-hydroxylated forms within macaque brain tissue sections.

a, c show two double bonds and **b, d** show one double bond in the molecular structure, exemplified in the brain regions GPi (**a, b**) and SNR (**c, d**).

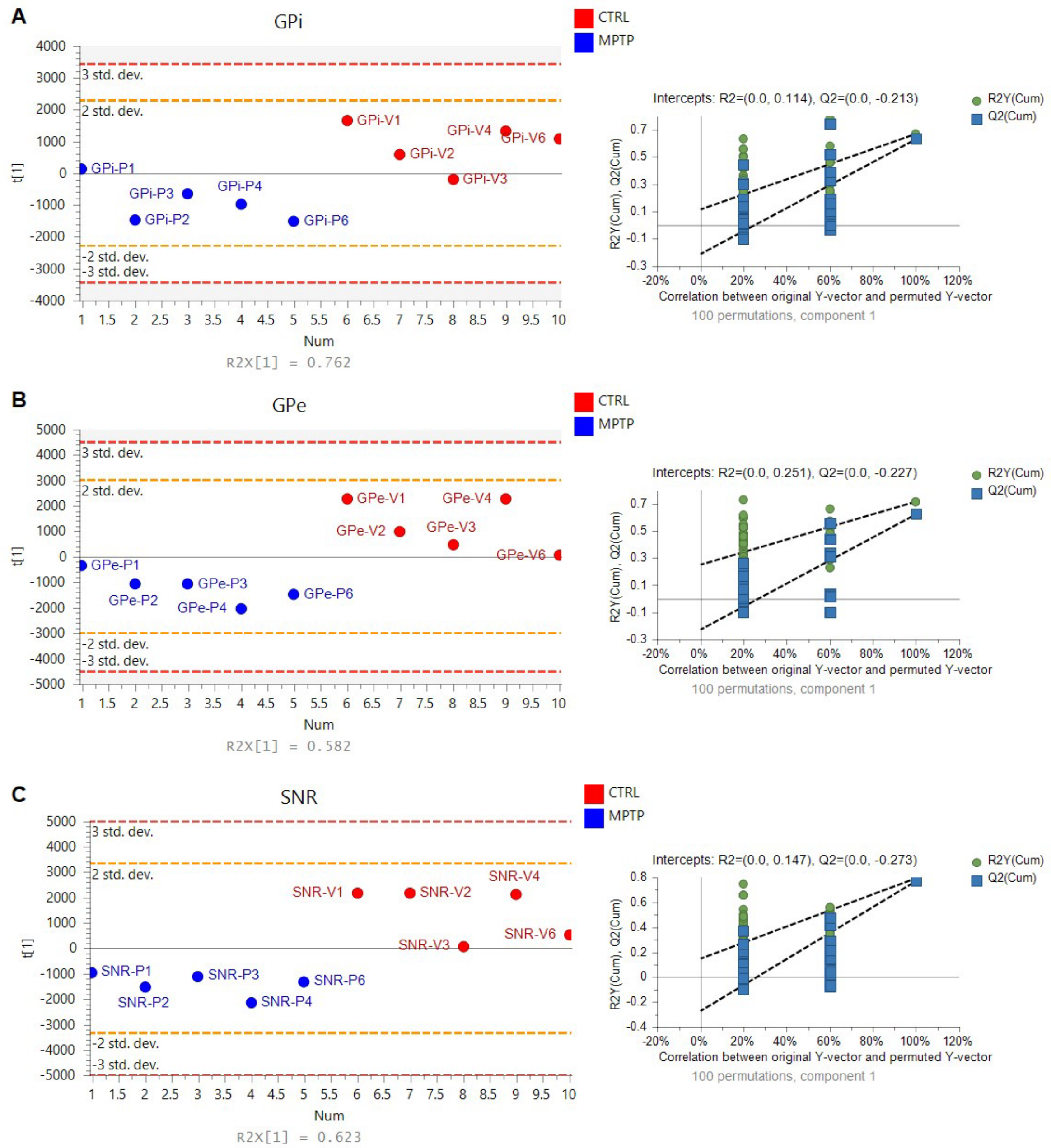

Supplementary Figure 4. Multivariate data analysis.

a Score plot of the one principal component PLS-DA model for MPTP-induced brain lipid alterations in the GPi region (left) and the corresponding validation permutation plot (right), R^2Y=0.669 and Q^2=0.630, *P* value of the cross-validated ANOVA= 0.03. **b** Score plot of the one principal component PLS-DA model for MPTP-induced brain lipid alterations in the GPe region (left) and the corresponding validation permutation plot (right), R^2Y=0.616 and Q^2=0.622, *P* value of the cross-validated ANOVA= 0.033. **c** Score plot of the one principal component PLS-DA model for MPTP-induced brain lipid alterations in the SNR region (left) and the corresponding validation permutation plot (right), R^2Y=0.794 and Q^2=0.770, *P* value of the cross-validated ANOVA= 0.006. Permutation tests calculated from 100 permutations showing the correlation between the original and permuted statistics (R^2Y. Q^2). The process included random re-ordering of the response variables (i.e., investigated classes), and the newly derived R^2Y and Q^2 are plotted against the degree of correlation between the permuted and original data. Significantly lower R^2Y and Q^2 values compared to the original values indicate the robustness of the model.

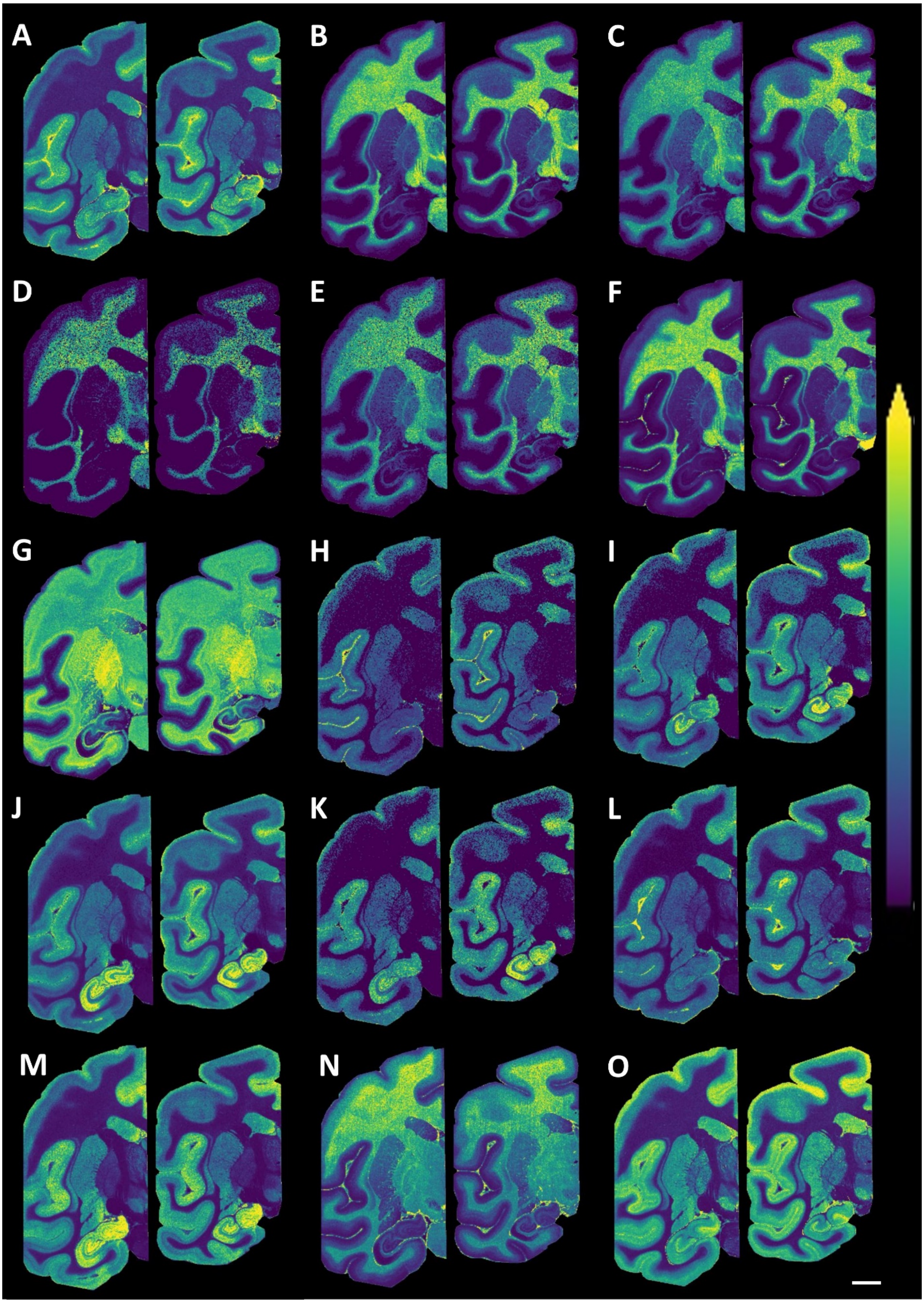
A
B
C
D
E
F
G
H
I
J
K
L
M
N
O

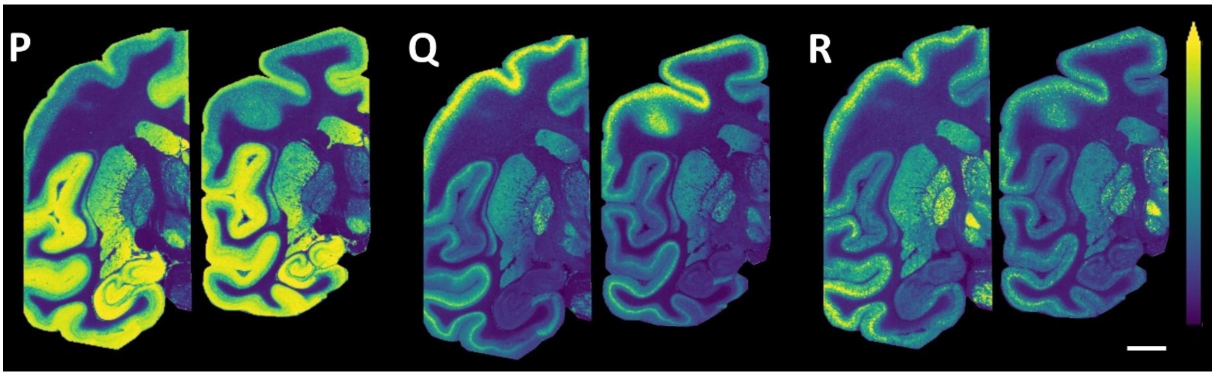

Supplementary Figure 5. Dual polarity MALDI-MSI reveals ion images of coronal brain tissue sections of macaque brains from control (left) and MPTP-lesioned monkeys (right).

The two groups reveal similar ion distribution images of several sphingolipids and glycerophospholipids including, **a** $[CerP\ (36:1)\text{-}H]^-$, **b** $[HexCer(d42:2)+K]^+$, **c** $[HexCer(t42:2)+K]^+$, **d** $[HexCer(d42:1)+K]^+$, **e** $[HexCer(t42:1)+K]^+$, **f** $[HexCer(d42:1)+K]^+$, **g** $[SHexCer(t42:1)\text{-}H]^-$, **h** $[GM3\ (36:1)\text{-}H]^-$, **i** $[GM2\ (36:1)\text{-}H]^-$, **j** $[GM1\ (36:1)\text{-}H]^-$, **k** $[GD1\ (36:1)+Na\text{-}2H]^-$, **l** $[PE\text{-}NMe_2\ (32:0)\text{-}H]^-$, **m** $[PE\ (38:4)\text{-}H]^-$, **n** $[PS\ (36:1)\text{-}H]^-$, **o** $[PI\ (36:4)\text{-}H]^-$, **p** $[PI\ (38:4)\text{-}H]^-$, **q** $[PI\ (40:6)\text{-}H]^-$, and **r** $[PC(40:6)+K]^+$. All ion images were RMS-normalized and scaled to the maximum intensity of each ion. Lateral resolution is 150 µm. Scale bar is 6 mm.

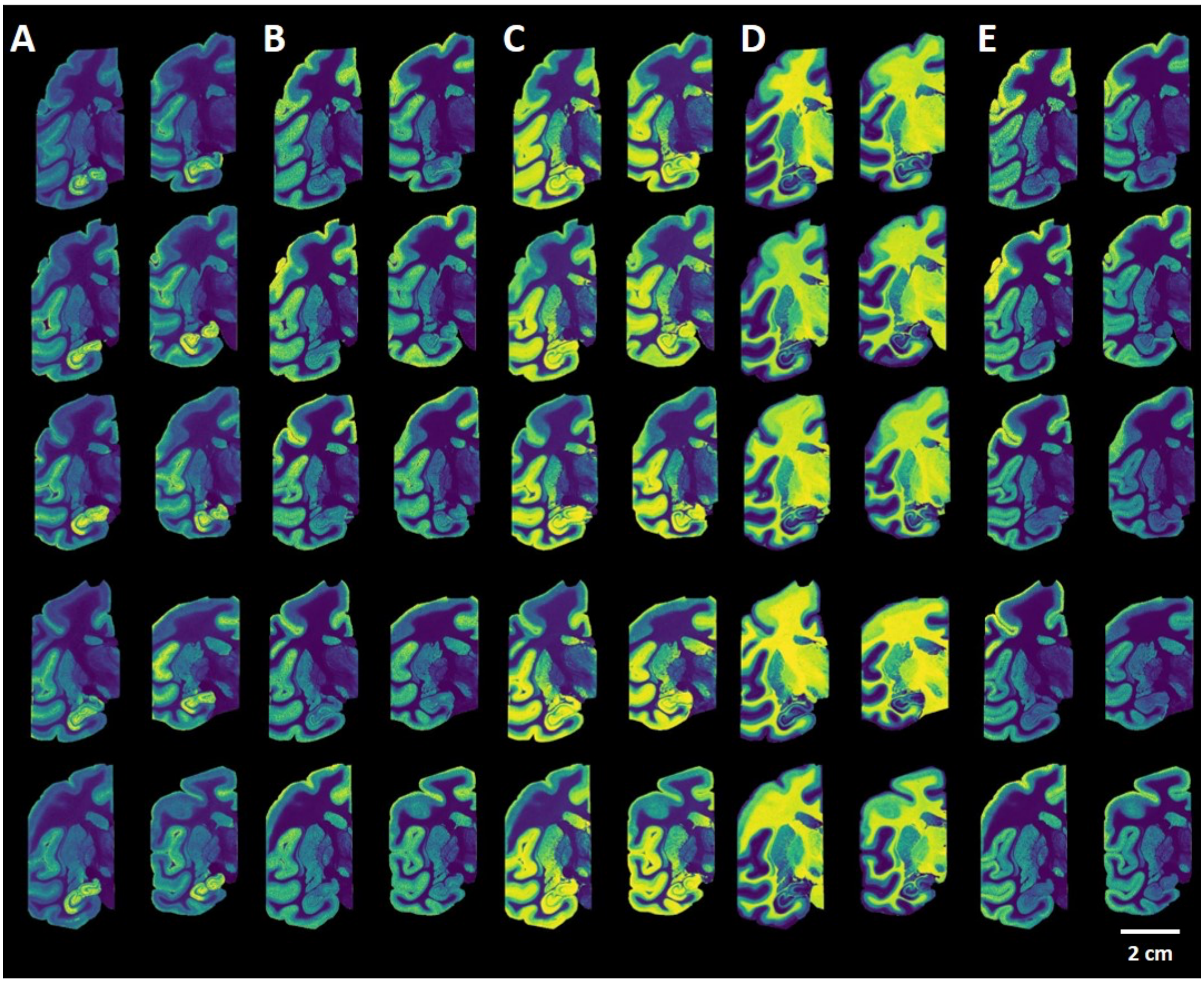

Supplementary Figure 6. Dual polarity MALDI-MSI reveals the some of the lipid distributions within the brain tissue sections of all the animals analyzed in this study.

The ion images of **a** [GM1 (36:1)-H]$^-$, **b** [PS (40:6)-H]$^-$, **c** [PE (40:6)-H]$^-$, **d** [ST (d42:2)-H]$^-$, and **e** [GD1+K-2H (36:1)-H]$^-$ in coronal brain tissue sections of all the control (n=5, left panel) and MPTP (n=5, right panel) macaque brain tissue sections. All ion distribution images are RMS normalized and are scaled to the maximum intensity of each individual ion. Lateral resolution is 150 µm.

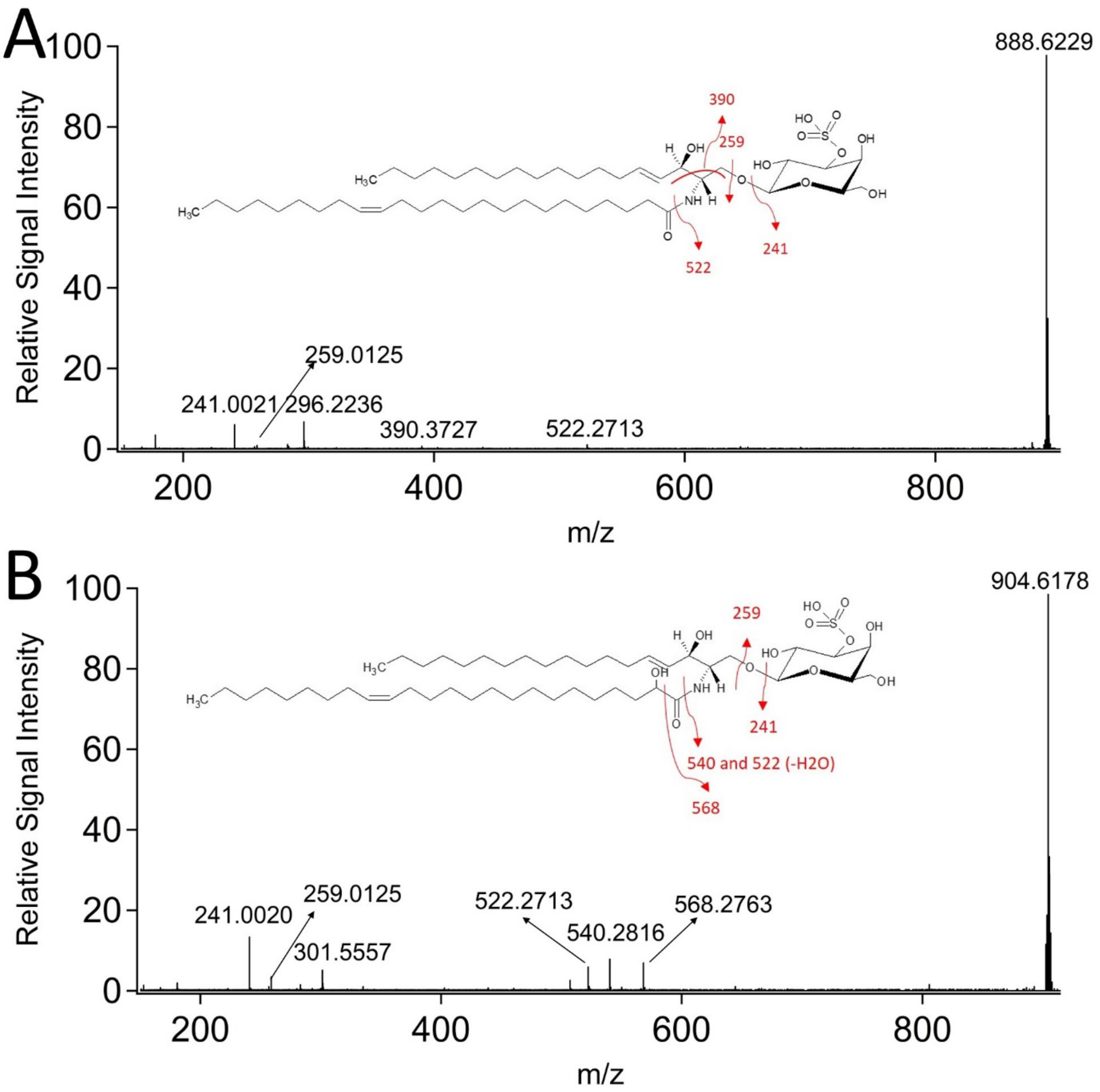

Supplementary Figure 7. MALDI-MS/MS spectra obtained from macaque brain tissue sections using MALDI-CID-FTICR MS/MS.

Precursor ions at **a** *m/z* 888.6229 and **b** *m/z* 904.6178. Product ions supporting the assignment of [SHexCer(d18:1/24:1)-H]$^-$ are found at *m/z* 241.0021 (loss of water from the sulfated hexose head group), *m/z* 259.0125 (product ion of the sulfated hexose head group), *m/z* 522.2713 (consecutive loss of 24:1 fatty acyl and water), *m/z* 390.3727 (related to loss of d18:1 long-chain base as $HCOCH=CH(CH_2)_{12}CH_3$).[1] Product ions supporting the assignment of [SHexCer(t18:1/24:1)-H]$^-$ are found at *m/z* 540.2816 (loss of 24:1 fatty acyl), *m/z* 522.2713 (consecutive loss of 24:1 fatty acyl and water), *m/z* 241.0021 (loss of water from the sulfated hexose head group), *m/z* 259.0125 (product ion of the sulfated hexose head group), and 568.2763 (the ion resulting from the cleavage of the same OC-CH(OH) bond to expel the fatty acyl moiety as an aldehyde).[1] Due to the isolation width of 1 *m/z* unit. additional isobaric and isomeric lipids may have fragmented, giving rise to additional product ions.

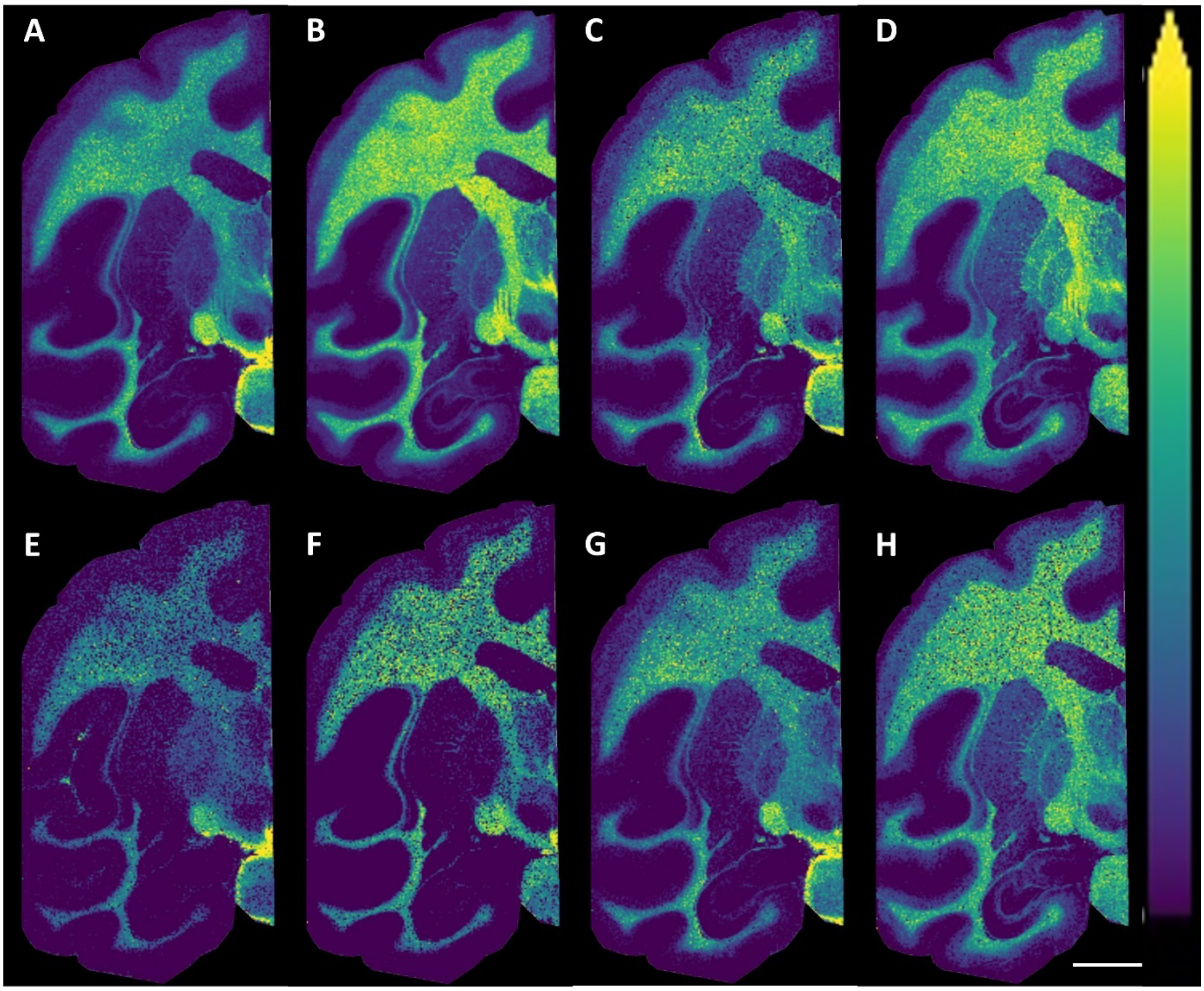

Supplementary Figure 8. MALDI-FTICR-MSI reveals similar distributions of several lipids.

Ion images show **a** [HexCer(d42:2)+Na]$^+$, **b** [HexCer(d42:2)+K]$^+$, **c** [HexCer(t42:2)+Na]$^+$, **d** [HexCer(t42:2)+K]$^+$, **e** [HexCer(d42:1)+Na]$^+$, **f** [HexCer(d42:1)+K]$^+$, **g** [HexCer(t42:1)+Na]$^+$, and **h** [HexCer(t42:1)+K]$^+$ in a control coronal macaque brain tissue section. All images were RMS-normalized and scaled to the maximum intensity of each ion. Lateral resolution is 150 µm. Scale bar is 6 mm.

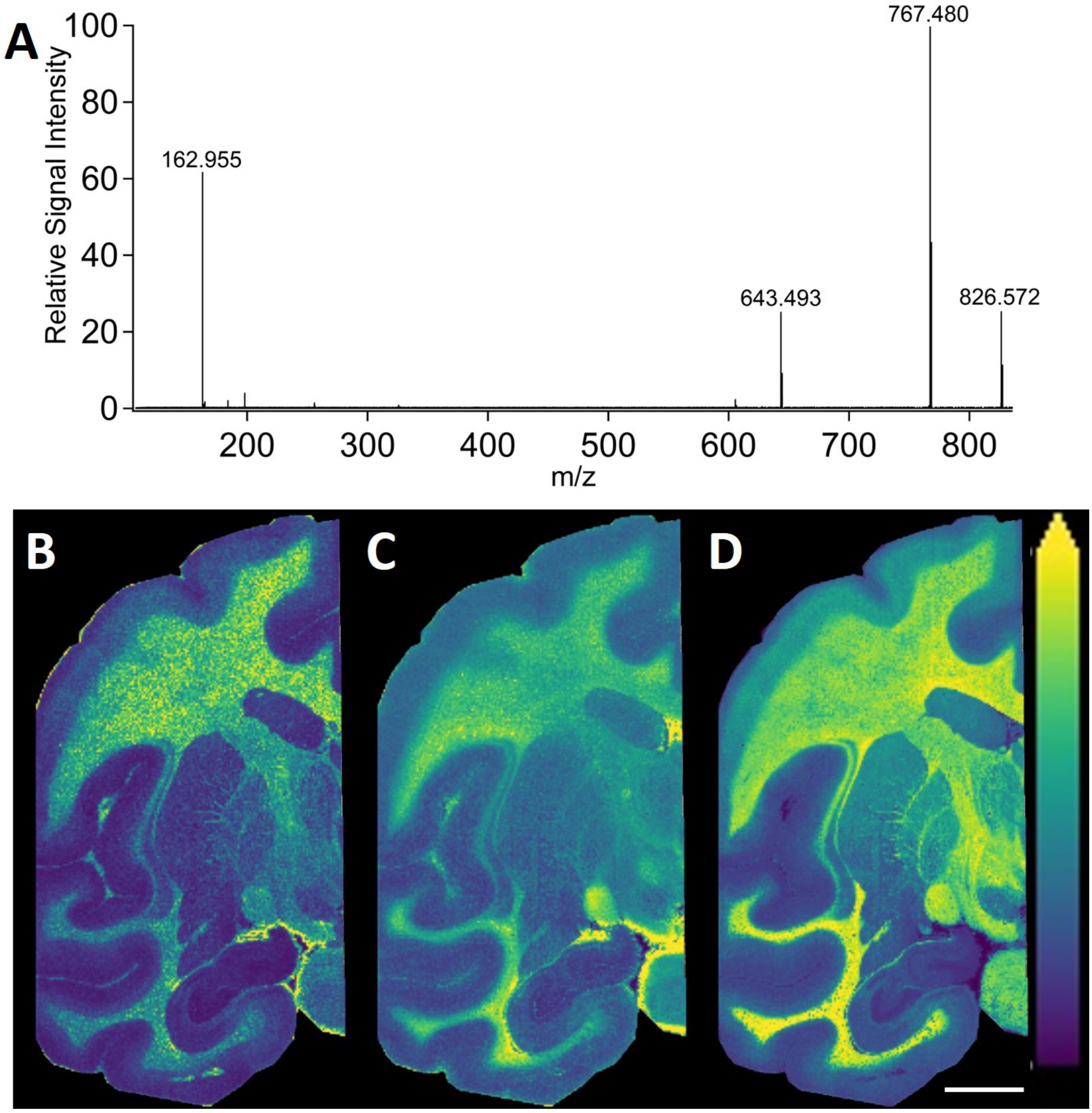

Supplementary Figure 9. MALDI-MS/MS spectra obtained from a coronal macaque brain tissue section using MALDI-CID-FTICR MS/MS.

Precursor ion at **a** *m/z* 826.572. Product ions supporting the assignment of $[PC(36:1)+K]^+$ are found at *m/z* 767.480 (loss of trimethylamine), *m/z* 643.493 (loss of phosphocholine head group), and *m/z* 162.955 (potassiated cyclophosphane). Due to the isolation width of 1 *m/z* unit, additional isobaric and isomeric lipids may have also fragmented giving rise to additional product ions. MALDI-FTICR-MSI reveals similar ion distribution images of **b** $[PC(36:1)+H]^+$, **c** $[PC(36:1)+Na]^+$, and **d** $[PC(36:1)+K]^+$ in a control coronal macaque brain tissue section. All images were RMS-normalized and scaled to the maximum intensity of each ion. The lateral resolution is 150 μm. Scale bar is 6 mm.

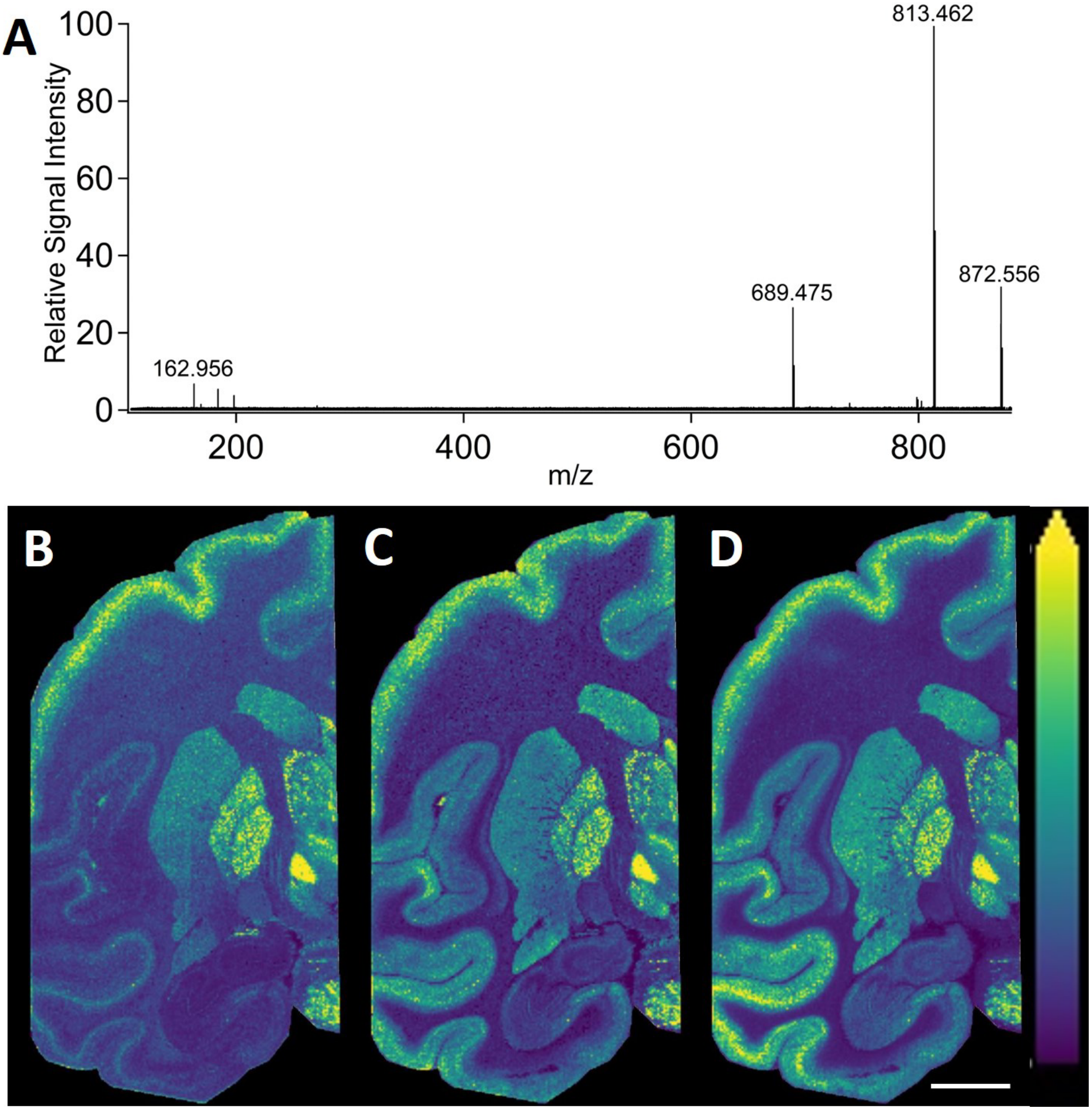

Supplementary Figure 10. MALDI-MS/MS spectra obtained from a coronal macaque brain tissue section using MALDI-CID-FTICR.

Precursor ions at **a** *m/z* 872.556. Product ions supporting the assignment of $[PC(40:6)+K]^+$ are found at *m/z* 813.462 (loss of trimethylamine), *m/z* 689.475 (loss of phosphocholine head group), and *m/z* 162.956 (potassiated cyclophosphane). Due to the isolation width of 1 *m/z* unit, additional isobaric and isomeric lipids may have also fragmented giving rise to additional product ions. MALDI-FTICR-MSI reveals similar ion distribution images of **b** $[PC(40:6)+H]^+$, **c** $[PC(40:6)+Na]^+$, and **d** $[PC(40:6)+K]^+$ in a control coronal macaque brain tissue section. All images were RMS-normalized and scaled to the maximum intensity of each ion. Lateral resolution is 150 µm. Scale bar is 6 mm.

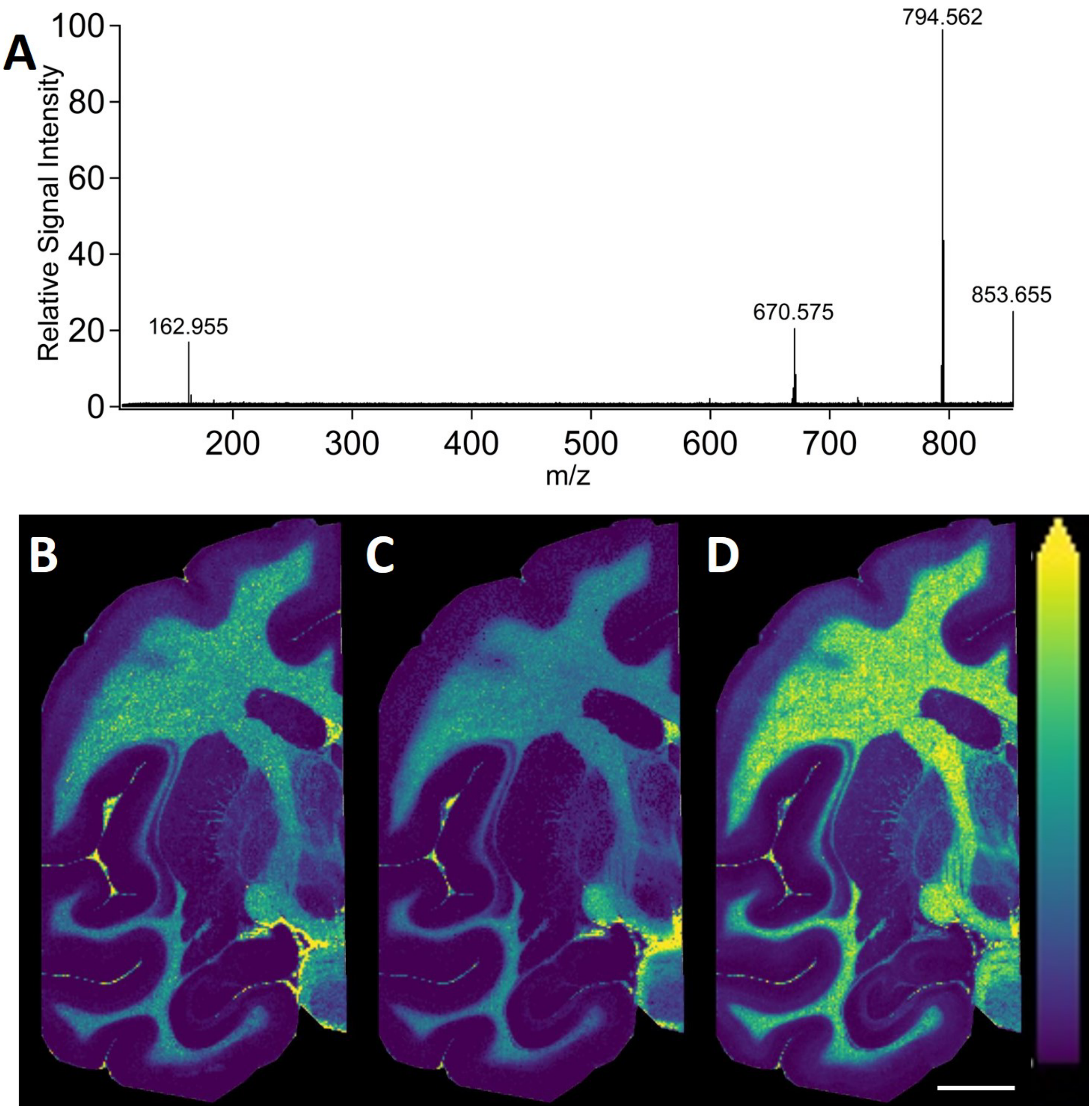

Supplementary Figure 11. MALDI-MS/MS spectra obtained from a coronal macaque brain tissue sections using MALDI-CID-FTICR MS/MS.

Precursor ions at **a** *m/z* 853.655. Product ions supporting the assignment of $[SM(42:1)+K]^+$ are found at *m/z* 794.562 (loss of trimethylamine), *m/z* 670.575 (loss of phosphocholine head group), and *m/z* 162.955 (sodiated cyclophosphane). Due to the isolation width of 1 *m/z* unit, additional isobaric and isomeric lipids may have also fragmented giving rise to additional product ions. MALDI-FTICR-MSI reveals similar ion distributions images of **b** $[SM(42:1)+H]^+$, **c** $[SM(42:1)+Na]^+$, and **d** $[SM(42:1)+K]^+$ in a control coronal macaque brain tissue section. All images were RMS-normalized and scaled to the maximum intensity of each ion. Lateral resolution is 150 µm. Scale bar is 6 mm.

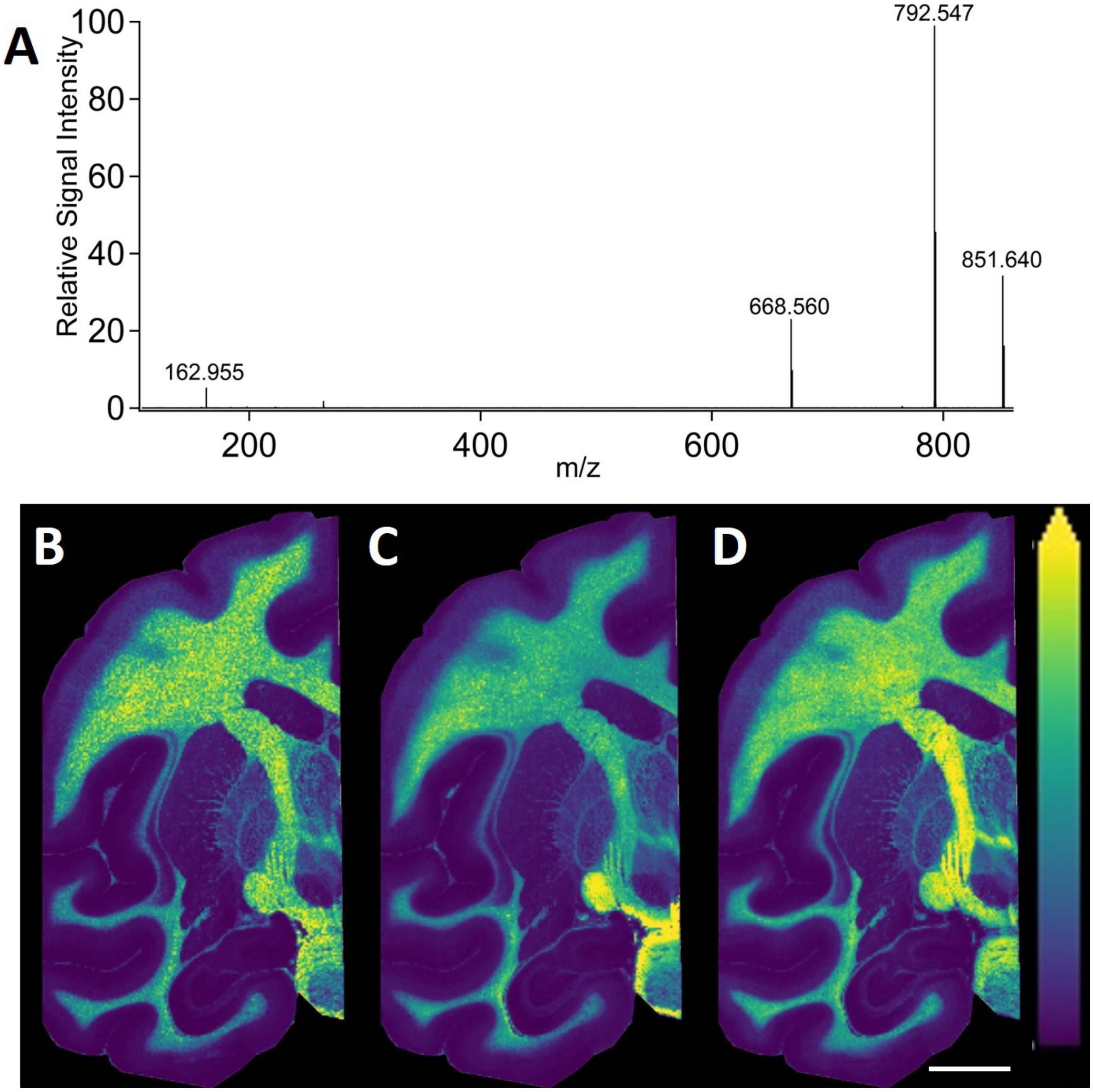

Supplementary Figure 12. MALDI-MS/MS spectra obtained from coronal macaque brain tissue sections using MALDI-CID-FTICR.

Precursor ions at **a** *m/z* 851.640. Product ions supporting the assignment of $[SM(42{:}2)+K]^+$ are found at *m/z* 792.547 (loss of trimethylamine), *m/z* 668.560 (loss of phosphocholine head group), and *m/z* 162.955 (sodiated cyclophosphane). Due to the isolation width of 1 *m/z* unit, additional isobaric and isomeric lipids may have also fragmented giving rise to additional product ions. MALDI-FTICR-MSI reveals similar ion distributions images of **b** $[SM(42{:}2)+H]^+$, **c** $[SM(42{:}2)+Na]^+$, and **d** $[SM(42{:}2)+K]^+$ in a control coronal macaque brain tissue section. All images were RMS-normalized and scaled to the maximum intensity of each ion. Lateral resolution is 150 μm. Scale bar is 6 mm.

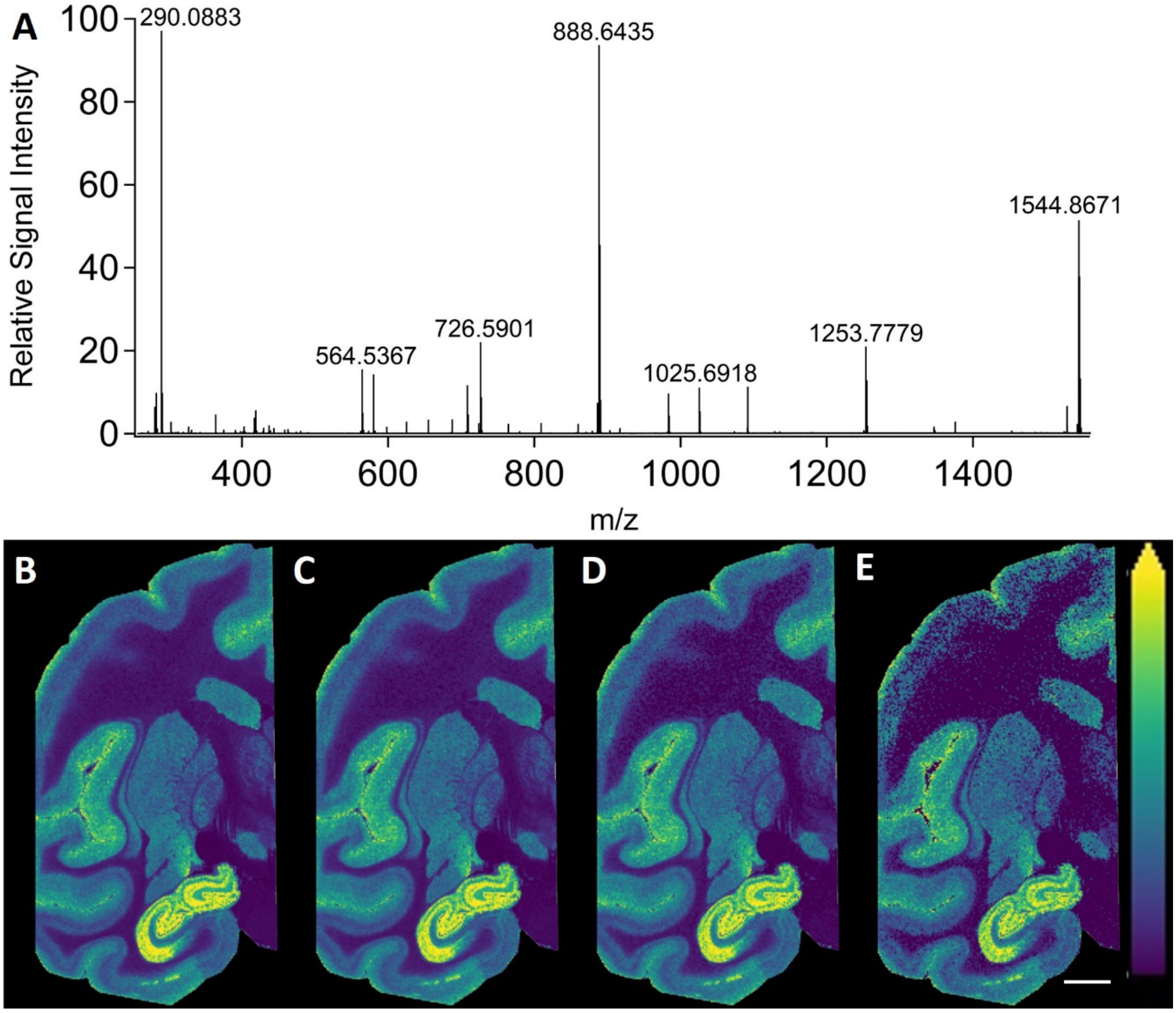

Supplementary Figure 13. MALDI-MS/MS spectra obtained from coronal macaque brain tissue sections using MALDI-CID-FTICR MS/MS.

Precursor ion at **a** *m/z* 1544.8671. Product ions supporting the assignment of [GM1(36:1)-H]$^-$ are found at *m/z* 1253.7779 (loss of sialic acid from the precursor ion), *m/z* 888.6435 (loss of sialic acid, one hexose and N-Acetylgalactosamine), *m/z* 726.5961 (loss of sialic acid. two hexoses and N-Acetylgalactosamine), *m/z* 564.5367 (loss of sialic acid. three hexoses and N-Acetylgalactosamine), and *m/z* 290.0883 (sialic acid). Due to the isolation width of 5 *m/z* unit, additional isobaric and isomeric lipids may have also fragmented giving rise to additional product ions. MALDI-FTICR-MSI reveals similar ion distribution images of the **b** first, **c** second, **d** third, and **e** fourth isotopic peak of [GM1(36:1)-H]$^-$ ion in a control coronal macaque brain tissue section. All images were RMS-normalized and scaled to the maximum intensity of each ion. Lateral resolution is 150 µm. Scale bar is 6 mm.

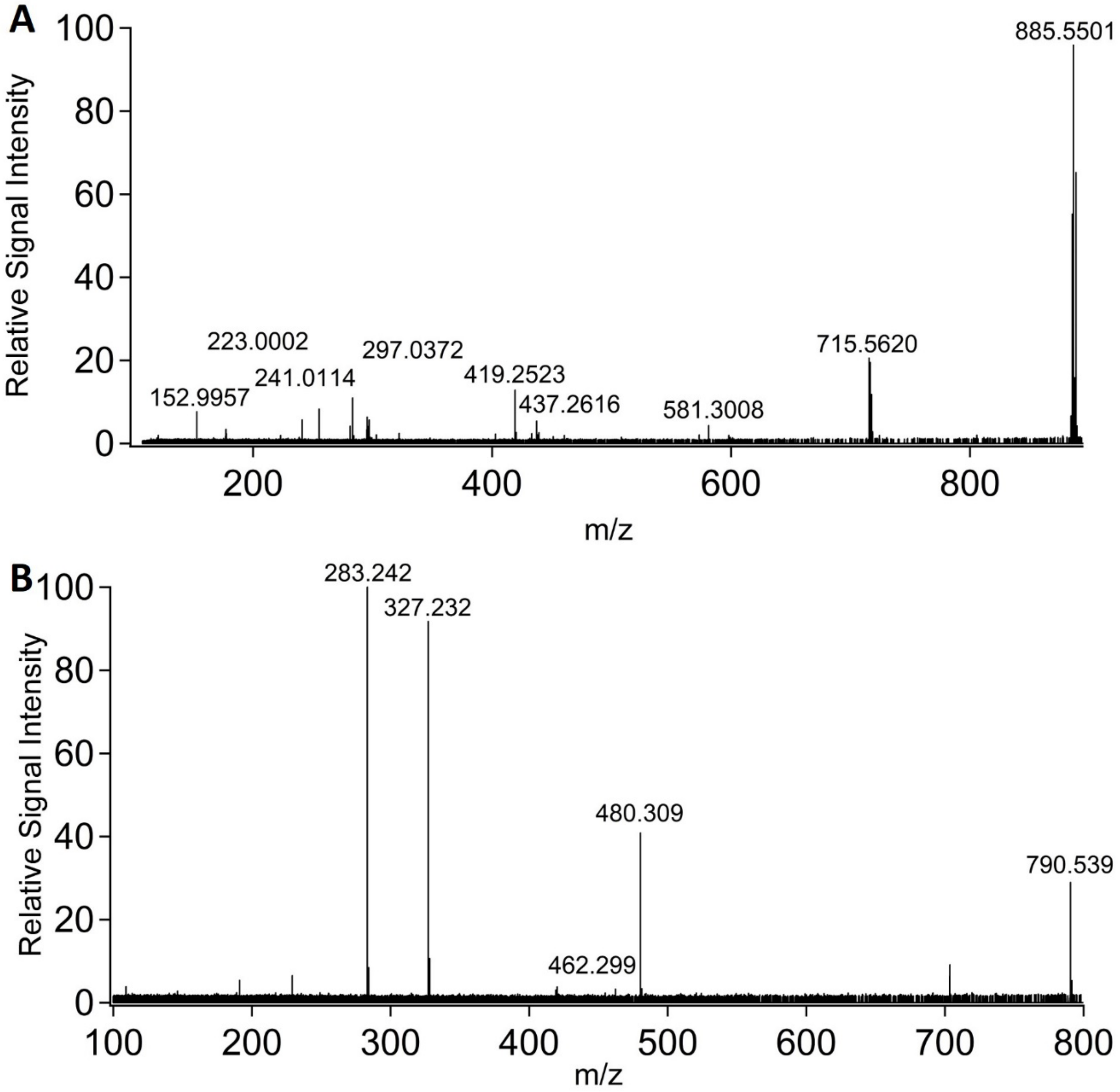

Supplementary Figure 14. MALDI-MS/MS spectra obtained from macaque brain tissue sections using MALDI-CID-FTICR MS/MS.

Precursor ions at **a** *m/z* 885.5501 and **b** *m/z* 790.539. Product ions supporting the assignment of [PI (18:0/20:4)-H]$^-$ are found at *m/z* 581.3008 (loss of sn2 acyl chain as ketene, RCH=C=O), *m/z* 419.2523 (neutral loss of sn2 RCOOH group and inositol), *m/z* 297.0372 (glycerophosphoinositol-2H_2O), *m/z* 223.0012 (inositol phosphate ion- 2H_2O), and *m/z* 152.9959 (glycerol-3-phosphate ion with loss of H_2O). Product ions supporting the assignment of [PE (18:0/22:6)-H]$^-$ are found at *m/z* 480.309 (loss of sn2 acyl chain as ketene, RCH=C=O), *m/z* 462.299 (neutral loss of sn2 RCOOH group), *m/z* 327.232 (sn2 RCOO$^-$ ion), and *m/z* 283.242 (sn1 RCOO$^-$ ion). Due to the isolation width of 1 *m/z* unit, additional isobaric and isomeric lipids may have also fragmented giving rise to additional product ions.

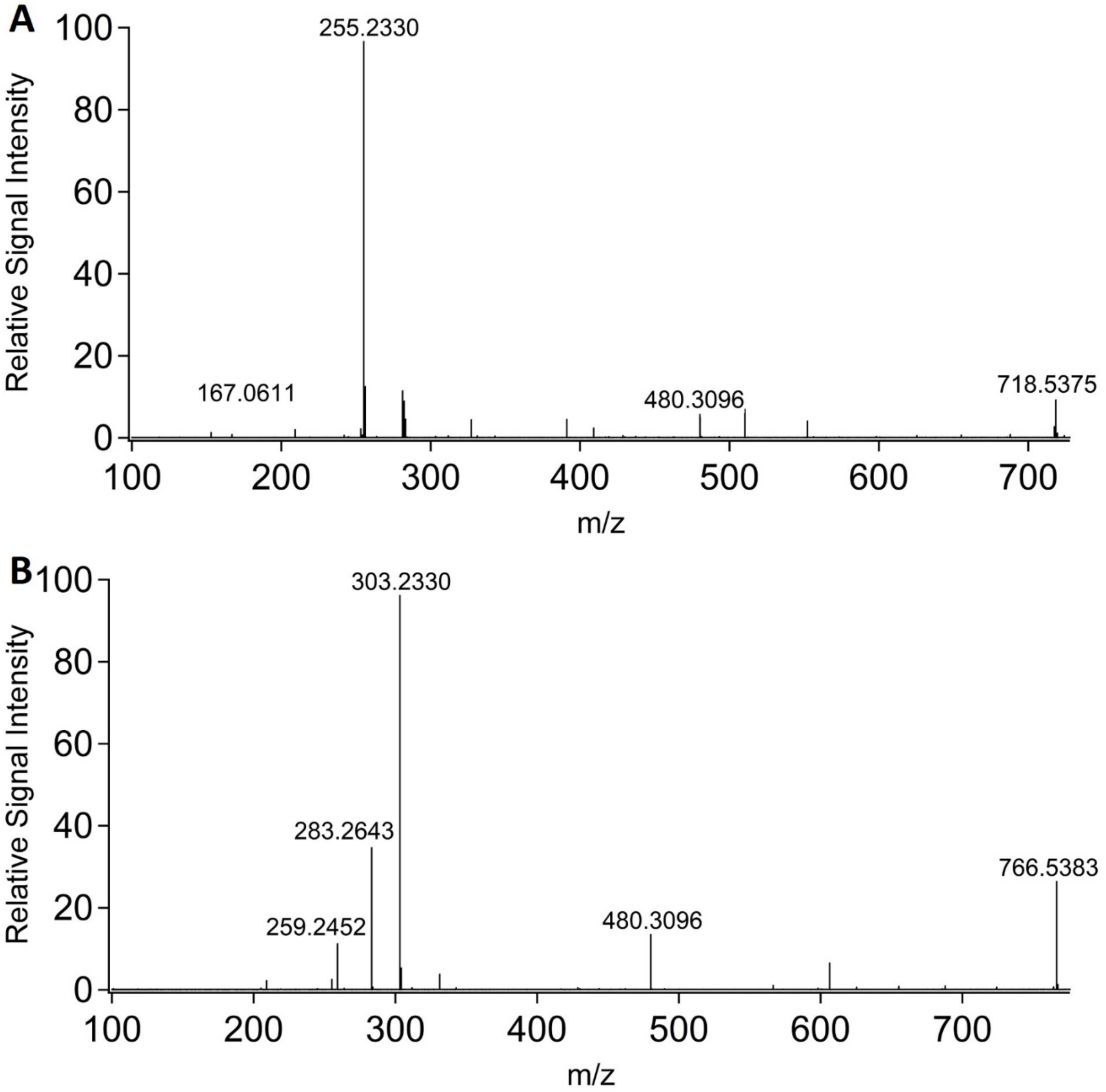

Supplementary Figure 15. MALDI-MS/MS spectra obtained from macaque brain tissue sections using MALDI-CID-FTICR.

Precursor ions at **a** *m/z* 718.5375 and **b** *m/z* 766.5383. Product ions supporting the assignment of [PE-NMe_2(16:0/16:0)-H]$^-$ are found at *m/z* 480.3096 (loss of sn2 acyl chain as ketene, RCH=C=O) from precursor ion), *m/z* 255.2330 (sn1 $RCOO^-$ ion), *m/z* 167.0611 (NMe_2-containing phosphatidyl-ethanolamine head group). Product ions supporting the assignment of [PE (18:0/20:4)-H]$^-$ are found at *m/z* 480.3096 (loss of sn2 acyl chain as ketene, RCH=C=O, from precursor ion), *m/z* 303.2330 (sn2 $RCOO^-$ ion), *m/z* 283.2643 (sn1 $RCOO^-$ ion), *m/z* 259.2452 (loss of CO_2 from sn2 $RCOO^-$ ion). **b** MALDI-MS/MS imaging of coronal mouse brain tissue section using MALDI-CID-FTICR reveals the distribution of product ions at *m/z* 480.3096 (loss of sn2 acyl chain as ketene, RCH=C=O) from precursor ion), *m/z* 303.2330 (sn2 $RCOO^-$ ion), *m/z* 283.2643 (sn1 $RCOO^-$ ion), and *m/z* 259.2452 (loss of CO_2 form sn2 $RCOO^-$ ion). Due to the isolation width of 1 *m/z* unit, additional isobaric and isomeric lipids may have also fragmented giving rise to additional product ions.

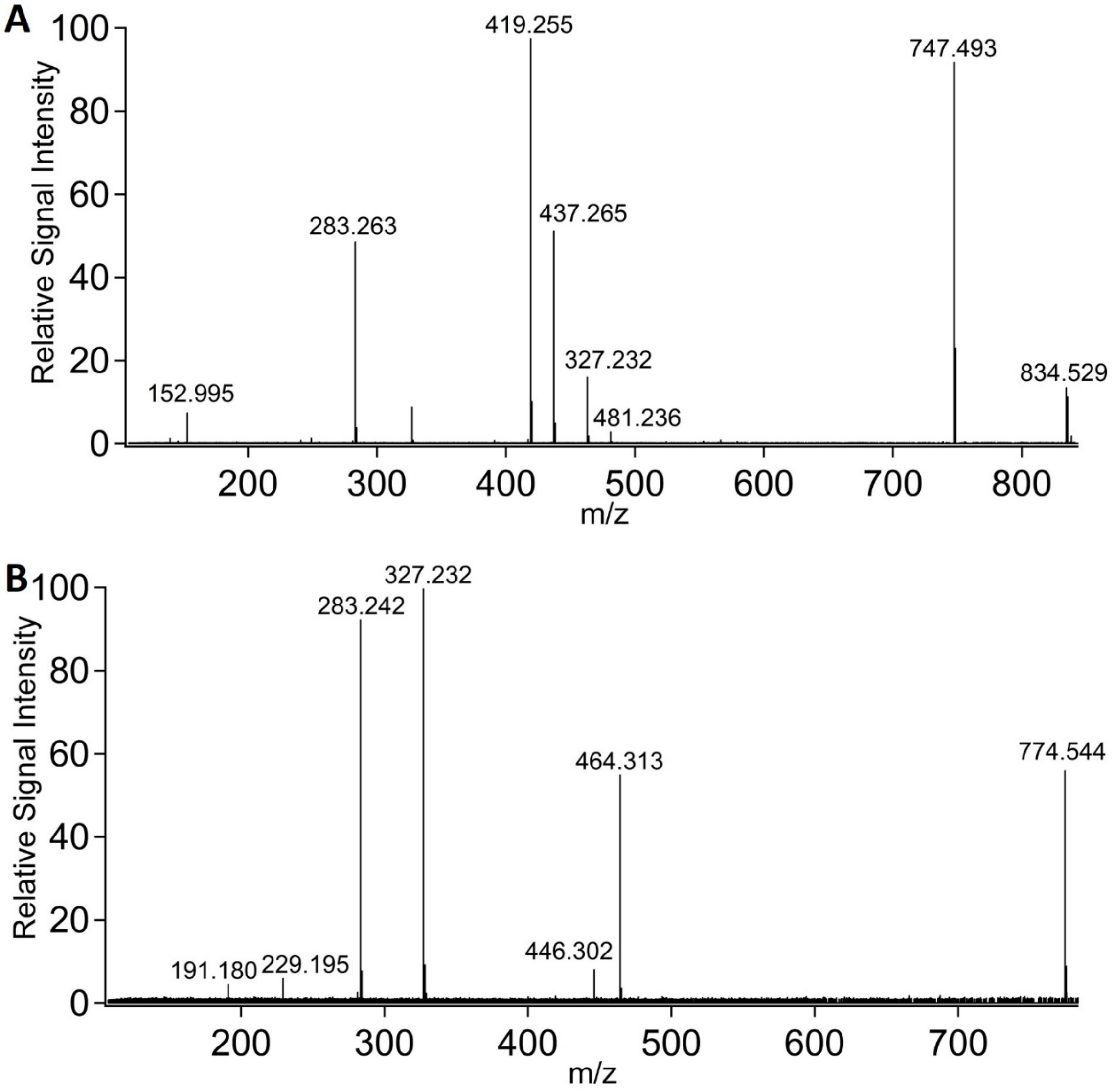

Supplementary Figure 16. MALDI-MS/MS spectra obtained from macaque brain tissue sections using MALDI-CID-FTICR.

Precursor ions at **a** *m/z* 834.529 and **b** *m/z* 774.544. Product ions supporting the assignment of [PS(18:0/22:6)-H]$^-$ are found at *m/z* 747.493 (loss of serine from precursor ion), *m/z* 481.236 (loss of sn1 acyl chain as ketene, RCH=C=O, and serine), *m/z* 437.2674 (loss of sn2 acyl chain as ketene, RCH=C=O, and serine), *m/z* 419.2566 (neutral loss of sn2 RCOOH group and serine), *m/z* 283.2642 (sn1 RCOO$^-$ ion), and *m/z* 152.995 (glycerol-3-phosphate ion with loss of H_2O). Product ions supporting the assignment of [PE(P-18:0/22:6)-H]$^-$ are found at *m/z* 464.313 (loss of sn2 acyl chain as ketene, RCH=C=O, from the precursor ion), *m/z* 446.302 (neutral loss of sn2 RCOOH group from the precursor ion), *m/z* 327.232 (sn2 RCOO- ion), and *m/z* 283.242 (loss of CO2 from sn2 RCOO- ion). Due to the isolation width of 1 *m/z* unit. additional isobaric and isomeric lipids may have also fragmented. giving rise to additional product ions.

Supplementary Table 1. Identified lipid species which showed significant differences in the control and MPTP-lesioned brain tissue samples.

Following optimization of the PLS-DA multivariate data analysis models, *m/z* values with high VIP scores were selected for identification in pathologically relevant brain regions (GPi, GPe, SNR). SHexCers are shown in bold font. Abbreviations: GPi, internal segment of globus pallidus; GPe, external segment of globus pallidus; SNR, substantia nigra pars reticulata.

GPi-VIP Scores	*m/z* value	Lipid Assignment
2.19051	904.619	**SHexCer(t42:2)**
1.75864	890.639	**SHexCer(d42:1)**
1.61006	905.624	**SHexCer(t42:2)-2ndisotope**
1.29293	918.636	**SHexCer(t43:2)**
1.27961	891.644	**SHexCer(d42:1)-2ndisotope**
1.01551	534.321	unknown
0.986171	890.603	**SHexCer(t41:2)**
0.942077	791.542	PE(40:6)-2ndisotope
0.915643	919.64	**SHexCer(t43:2)-2ndisotope**
0.802998	810.528	unknown
0.713286	891.604	**SHexCer(t41:2)-2ndisotope**
0.701061	507.292	unknown
0.646946	723.498	unknown
0.617032	876.624	**SHexCer(d41:0)**
0.613811	917.66	**SHexCer(d44:2)-2ndisotope**
0.564961	877.628	**SHexCer(d41:0)-2ndisotope**
0.561282	892.647	**SHexCer(d42:1)-3rdisotope**
0.558408	862.608	**SHexCer(d40:1)**

GPe-VIP Scores	*m/z* value	Lipid Assignment
1.93818	851.64	SM(42:2)+K
1.38601	904.619	**SHexCer(t42:2)**
1.32473	813.684	SM(42:2)
1.29488	852.644	SM(42:2)+K-2ndisotope
1.27472	826.572	PC(36:1)+K
1.16558	890.639	**SHexCer(d42:1)**
1.07499	905.624	**SHexCer(t42:2)-2ndisotope**
1.06681	918.636	**SHexCer(t43:2)**
1.03137	790.539	PE(40:6)
0.975902	814.688	SM(42:2)-2ndisotope
0.922982	834.529	PS(40:6)
0.853489	774.544	PE(P-40:6)
0.838396	836.669	SM(42:2)+Na-2ndisotope

0.824161	919.64	**SHexCer(t43:2)-2ndisotope**
0.754808	891.644	**SHexCer(d42:1)-2ndisotope**
0.747237	849.561	unknown
0.730099	811.602	PC(36:1)+Na-2ndisotope
0.651562	791.542	PE(40:6)-2ndisotope
0.604994	1648.97	unknown
0.586331	865.656	SM(43:2)+K

SNR-VIP Scores	*m/z* value	Lipid Assignment
1.66336	826.572	PC(36:1)+K
1.58814	851.64	SM(42:2)
1.37632	788.617	PC(36:1)
1.12923	852.644	SM(42:2)-2ndisotope
1.11069	827.576	PC(36:1)+K-2ndisotope
1.04744	904.619	**SHexCer(t42:2)**
0.890191	789.62	PC(36:1)-2ndisotope
0.861586	890.639	**SHexCer(d42:1)**
0.845522	905.624	**SHexCer(t42:2)-2ndisotope**
0.703227	891.644	**SHexCer(d42:1)-2ndisotope**
0.597751	918.636	**SHexCer(t43:2)**
0.572284	853.646	SM(42:2)-3rdisotope
0.500377	890.632	unknown

Supplementary Table 2. Results from statistical analysis of sulfatide lipids between the control and MPTP-treated groups using multiple t-test.

The results presented in bold indicate significant differences ($P \leq 0.05$) in long-chain non-hydroxylated and non-hydroxylated sulfatides in multiple brain regions. Abbreviations: PoG, postcentral gyrus; PrG, precentral gyrus; PCgG, posterior singulate gyrus; STh, subthalamic nucleus; STG, superior temporal gyrus; MTG, middle temporal gyrus; ITG, inferior temporal gyrus; Ent, entorhinal area; Hipp, hippocampus; Cd, caudate nucleus; Ins, insula; Cl, claustrum; Put, putamen; GP, globus pallidus; Th, thalamus; ic, internal capsule; LG, lateral geniculate nucleus; tw, temporal white matter; cw, cerebral white matter; SNR, substantia nigra pars reticulata; nd, not detected.

SHexCer(t42:2)	Significant	P value	SHexCer(t43:2)	Significant	P value
Cd	No	0.3067	Cd	No	0.2804
Clau	No	0.3671	Clau	No	0.4733
cw	No	0.3945	cw	No	0.1781
Ent	No	0.1767	Ent	No	0.1682
GPe	No	0.0579	**GPe**	**Yes**	**0.0166**
**GPi**	**Yes**	**0.0175**	**GPi**	**Yes**	**0.0193**
Hip	No	0.1159	Hip	No	0.1364
Ic	No	0.4563	ic	No	0.3306
Ins	No	0.4704	Ins	No	0.1958
ITG	No	0.128	ITG	No	0.1093
MTG	No	0.2119	MTG	No	0.0944
opt	No	0.7963	opt	No	0.5772
PCgG	No	0.1873	PCgG	No	0.0772
PoG	No	0.2416	PoG	No	0.1139
PrG	No	0.5848	PrG	No	0.1327
Put	No	0.0884	Put	No	0.0833
**SNR**	**Yes**	**0.0246**	**SNR**	**Yes**	**0.0176**
STG	No	0.4385	STG	No	0.1805
Tha	No	0.2734	Tha	No	0.1316
tw	No	0.1398	tw	No	0.1539
STh	No	0.278	STh	No	0.2044

SHexCer(d41:1)	Significant	P value	SHexCer(d42:1)	Significant	P value
Cd	No	0.6043	Cd	No	0.6918
Clau	No	0.1893	Clau	No	0.1331
cw	No	0.9064	cw	No	0.2033
Ent	No	0.3885	**Ent**	**Yes**	**0.0192**
GPe	No	0.1807	**GPe**	**Yes**	**0.0268**
**GPi**	**Yes**	**0.0269**	**GPi**	**Yes**	**0.0089**
Hip	No	0.0527	Hip	No	0.1749
ic	No	0.8206	**ic**	**Yes**	**0.0366**
Ins	No	0.6078	**Ins**	**Yes**	**0.0047**
ITG	No	0.8368	ITG	No	0.2261
MTG	No	0.1329	**MTG**	**Yes**	**0.0094**
opt	No	0.5103	opt	No	0.1156
PCgG	No	0.7973	PCgG	No	0.2317

PoG	No	0.441	**PoG**	**Yes**	**0.0281**
PrG	No	0.8328	PrG	No	0.3431
Put	No	0.2629	Put	No	0.0852
**SNR**	**Yes**	**0.0252**	SNR	No	0.0501
**STG**	**Yes**	**0.0378**	STG	No	0.5386
Tha	No	0.5673	Tha	No	0.627
tw	No	0.1808	tw	No	0.2025
STh	No	0.1644	**STh**	**Yes**	**0.0197**

SHexCer(t42:3)	Significant	P value	SHexCer(t41:2)	Significant	P value
Cd	No	0.247	Cd	No	0.7417
Clau	No	0.213	Clau	No	0.9473
cw	No	0.2066	cw	No	0.9695
Ent	No	0.3711	Ent	No	0.3671
GPe	No	0.0606	**GPe**	**Yes**	**0.0499**
GPi	No	0.0598	GPi	No	0.557
Hip	No	0.3416	Hip	No	0.3544
ic	No	0.2553	ic	nd	nd
Ins	No	0.3457	Ins	No	0.3974
ITG	No	0.249	ITG	No	0.3357
MTG	No	0.3738	MTG	No	0.381
opt	No	0.9744	opt	nd	nd
PCgG	No	0.5528	PCgG	No	0.2908
PoG	No	0.5817	PoG	No	0.3869
PrG	No	0.0682	PrG	No	0.3631
Put	No	0.1039	Put	No	0.4862
**SNR**	**Yes**	**0.0429**	SNR	No	0.3351
STG	No	0.4842	STG	No	0.3542
Tha	No	0.1217	Tha	No	0.1892
tw	No	0.2159	tw	No	0.0745
STh	No	0.1579	STh	No	0.3076

SHexCer(d40:1)	Significant	P value	SHexCer(d40:2)	Significant	P value
Cd	No	0.4842	Cd	No	0.1201
**Clau**	**Yes**	**0.0273**	**Clau**	**Yes**	**0.0392**
**cw**	**Yes**	**0.0337**	cw	No	0.0605
Ent	No	0.2481	Ent	No	0.1654
**GPe**	**Yes**	**0.0236**	**GPe**	**Yes**	**0.0104**
**GPi**	**Yes**	**0.0043**	GPi	No	0.0665
Hip	No	0.4363	Hip	No	0.7983
**ic**	**Yes**	**0.0293**	ic	No	0.2451
Ins	No	0.1621	**Ins**	**Yes**	**0.0237**
ITG	No	0.1791	ITG	No	0.6566
MTG	No	0.0595	**MTG**	**Yes**	**0.0496**
opt	No	0.0835	**opt**	**Yes**	**0.0471**
PCgG	No	0.1469	**PCgG**	**Yes**	**0.0449**
**PoG**	**Yes**	**0.0083**	**PoG**	**Yes**	**0.001**
**PrG**	**Yes**	**0.0059**	**PrG**	**Yes**	**0.0155**

**Put**	**Yes**	**0.0058**	Put	No	0.3359
SNR	No	0.0586	SNR	No	0.1792
**STG**	**Yes**	**0.0364**	STG	No	0.1347
Tha	No	0.111	Tha	No	0.3461
tw	No	0.0646	tw	No	0.2191
**STh**	**Yes**	**0.0249**	STh	No	0.278

Supplementary Table 3. List of assigned lipid species with high mass accuracy obtained from MALDI-FTICR-MSI experiments.

A search of the *m/z* values with a 0.01 *m/z* mass tolerance in LIPID MAPS was conducted for both negative and positive polarities including all the ion types. Lipid species marked with two asterisks (**) were identified using mass accuracy, MS/MS, isotopic distribution, and/or by comparing the observed distributions of different adducts of the same molecule across the coronal brain tissue sections. Sulfatide species marked with one asterisk (*) were identified solely based on the *m/z* 241.0021 product ion (loss of water from the sulfated hexose head group).[1] Ganglioside species were identified based on the product ion at *m/z* 290.0883 (sialic acid) and the resulting ion from the loss of sialic acid from the precursor ions.[2] Lipids that are not marked with an asterisk (*) were identified based solely on mass accuracy since the peak intensity was too low for MS/MS analysis of the tissue. For ion species where specific information about the fatty acid chains of the lipids was obtained, this information is included in the annotations. For species identified solely based on head group fragments or mass accuracy, a combined isomer annotation was provided.

Lipid Species Assignment	Ion Type	Formula	*m/z* theoretical	*m/z* observed	ppm error
SHexCer(d30:2)	$[M-H]^-$	C36H67NO11S	720.436207	720.43655	0.476
SHexCer(t30:2)	$[M-H]^-$	C36H67NO12S	736.431121	736.43123	0.148
SHexCer(d32:1)	$[M-H]^-$	C38H73NO11S	750.483157	750.48336	0.270
SHexCer(t32:1)	$[M-H]^-$	C38H73NO12S	766.478072	766.47856	0.637
SHexCer(d32:2)	$[M-H]^-$	C38H71NO11S	748.467507	748.46773	0.298
SHexCer(t32:2)	$[M-H]^-$	C38H71NO12S	764.462421	764.46306	0.836
SHexCer(d34:2)	$[M-H]^-$	C40H75NO11S	776.498807	776.49902	0.274
SHexCer(t34:2)	$[M-H]^-$	C40H75NO12S	792.493722	792.49408	0.452
SHexCer(d36:2)	$[M-H]^-$	C42H79NO11S	804.530107	804.5309	0.986
SHexCer(t36:2)	$[M-H]^-$	C42H79NO12S	820.525022	820.52555	0.643
SHexCer(d36:1)*	$[M-H]^-$	C42H81NO11S	806.545757	806.54651	0.934
SHexCer(t18:1/18:0)**	$[M-H]^-$	C42H81NO12S	822.540672	822.54114	0.569
SHexCer(d38:2)	$[M-H]^-$	C44H83NO11S	832.561407	832.56154	0.160
SHexCer(t38:2)	$[M-H]^-$	C44H83NO12S	848.556322	848.55691	0.693
SHexCer(d38:1)	$[M-H]^-$	C44H85NO11S	834.577057	834.57728	0.267
SHexCer(t38:1)	$[M-H]^-$	C44H85NO12S	850.571972	850.57169	-0.332
SHexCer(d40:2)*	$[M-H]^-$	C46H87NO11S	860.592707	860.59299	0.329
SHexCer(d18:1:h22:1)**	$[M-H]^-$	C46H87NO12S	876.587622	876.58799	0.420
SHexCer(d40:1)*	$[M-H]^-$	C46H89NO11S	862.608357	862.60792	-0.507
SHexCer(d18:1/h22:0)**	$[M-H]^-$	C46H89NO12S	878.603272	878.60335	0.089
SHexCer(d18:1:h23:1)**	$[M-H]^-$	C47H89NO12S	890.603272	890.60422	1.064
SHexCer(d41:2)*	$[M-H]^-$	C47H89NO11S	874.608357	874.60804	-0.362
SHexCer(d18:1:23:0)**	$[M-H]^-$	C47H91NO11S	876.624007	876.62448	0.540

SHexCer(d18:1:h23:0)**	$[M-H]^-$	C47H91NO12S	892.618922	892.61902	0.110
SHexCer(t42:3)	$[M-H]^-$	C48H89NO12S	902.603272	902.60402	0.829
SHexCer(d42:3)*	$[M-H]^-$	C48H89NO11S	886.608357	886.60911	0.849
SHexCer(d18:1/24:1)**	$[M-H]^-$	C48H91NO11S	888.624007	888.62402	0.015
SHexCer(d18:1/h24:1)**	$[M-H]^-$	C48H91NO12S	904.618922	904.61979	0.960
SHexCer(d18:1/24:0)**	$[M-H]^-$	C48H93NO11S	890.639658	890.63995	0.328
SHexCer(d18:1/h24:0)**	$[M-H]^-$	C48H93NO12S	906.634572	906.63464	0.075
SHexCer(d18:1/25:1)**	$[M-H]^-$	C49H93NO11S	902.639658	902.63995	0.323
SHexCer(d18:1/h25:1)**	$[M-H]^-$	C49H93NO12S	918.634572	918.63546	0.967
SHexCer(d44:3)	$[M-H]^-$	C50H93NO11S	914.639658	914.6408	1.249
SHexCer(d18:1/h26:2)**	$[M-H]^-$	C50H93NO12S	930.634572	930.63601	1.545
SHexCer(d18:1/26:1)**	$[M-H]^-$	C50H95NO11S	916.655308	916.65554	0.253
SHexCer(d18:1/h26:1)**	$[M-H]^-$	C50H95NO12S	932.650222	932.65091	0.738
SHexCer(d44:1)	$[M-H]^-$	C50H97NO11S	918.670958	918.67157	0.666
SHexCer(d18:1/h26:0)**	$[M-H]^-$	C50H97NO12S	934.665872	934.66671	0.897
SHexCer(d18:1/h25:0)**	$[M-H]^-$	C49H95NO12S	920.650222	920.65095	0.791
HexCer(d42:2)**	$[M+K]^+$	C48H91NO8K	848.637627	848.63835	0.852
HexCer(d42:2)**	$[M+Na]^+$	C48H91NO8Na	832.66369	832.66405	0.432
HexCer(t42:2)**	$[M+K]^+$	C48H91NO9K	864.632541	864.63309	0.635
HexCer(t42:2)**	$[M+Na]^+$	C48H91NO9Na	848.658604	848.65908	0.561
HexCer(d42:1)**	$[M+K]^+$	C48H93NO8K	850.653277	850.65255	-0.855
HexCer(d42:1)**	$[M+Na]^+$	C48H93NO8Na	834.67934	834.67866	-0.815
HexCer(t42:1)**	$[M+K]^+$	C48H93NO9K	866.648191	866.64797	-0.255
HexCer(t42:1)**	$[M+Na]^+$	C48H93NO9Na	850.674254	850.67347	-0.922
SM(42:2)**	$[M+H]^+$	C47H93N2O6P	813.684402	813.68441	0.010
SM(42:2)**	$[M+Na]^+$	C47H93N2NaO6P	835.666346	835.66608	-0.318
SM(42:2)**	$[M+K]^+$	C47H93N2KO6P	851.640284	851.64032	0.042
SM(42:1)**	$[M+H]^+$	C47H95N2O6P	815.700052	815.70011	0.071
SM(42:1)**	$[M+Na]^+$	C47H95N2NaO6P	837.681996	837.68172	-0.329
SM(42:1)**	$[M+K]^+$	C47H95N2KO6P	853.655934	853.65591	-0.028
GM3(38:1)**	$[M-H]^-$	C61H112N2O21	1207.768482	1207.76851	0.023
GM1(38:1)**	$[M-H]^-$	C75H135N3O31	1572.900678	1572.90083	0.097
GD1(38:1)**	$[M+Na-2H]^-$	C86H152N4O39Na	1885.978039	1885.97617	-0.991
GM1(36:1)**	$[M-H]^-$	C73H131N3O31	1544.869378	1544.87053	0.746
GM2(36:1)**	$[M-H]^-$	C67H121N3O26	1382.816555	1382.81548	-0.777
GM3(36:1)**	$[M-H]^-$	C59H108N2O21	1179.737182	1179.73654	-0.544

GD1(38:1)**	$[M+K-2H]^-$	C86H152N4O39K	1901.951976	1901.95025	-0.907
GD1(36:1)**	$[M+K-2H]^-$	C84H148N4O39Na	1873.92167	1873.920676	-0.530
GD1(36:1)**	$[M+Na-2H]^-$	C84H148N4O39Na	1857.946739	1857.94715	0.221
GD1(36:1)**	$[M-H]^-$	C84H148N4O39	1835.964794	1835.96594	0.624
GD1(38:1)**	$[M-H]^-$	C86H152N4O39	1863.996095	1863.99582	-0.148
PS(18:0/18:1)**	$[M-H]^-$	C42H80NO10P	788.544708	788.54545	0.941
PS(18:0/22:6)**	$[M-H]^-$	C46H78NO10P	834.529058	834.52951	0.542
PE(16:0/22:6)**	$[M-H]^-$	C43H74NO8P	762.507929	762.50832	0.513
PE(18:0/22:6)**	$[M-H]^-$	C45H78NO8P	790.539229	790.53957	0.431
PI(16:0/20:4)**	$[M-H]^-$	C45H79O13P	857.518553	857.51821	-0.400
PI(18:0/20:4)**	$[M-H]^-$	C47H83O13P	885.549853	885.55034	0.550
PI(18:0/22:6)**	$[M-H]^-$	C49H83O13P	909.549853	909.55078	1.019
PE-NMe2(16:0/16:0)**	$[M-H]^-$	C39H78NO8P	718.539229	718.53966	0.600
PE(18:0/20:4)**	$[M-H]^-$	C43H78NO8P	766.539229	766.53977	0.706
PE(P-18:0/22:6)**	$[M-H]^-$	C45H78NO7P	774.544314	774.54482	0.653
PC(36:1)**	$[M+H]^+$	C44H86NO8P	788.616382	788.61641	0.036
PC(36:1)**	$[M+Na]^+$	C44H86NO8PNa	810.598326	810.59894	0.757
PC(36:1)**	$[M+K]^+$	C44H86NO8PK	826.572264	826.57289	0.757
PC(40:6)**	$[M+H]^+$	C48H84NO8P	834.600732	834.60127	0.645
PC(40:6)**	$[M+Na]^+$	C48H84NO8PNa	856.582676	856.58277	0.110
PC(40:6)**	$[M+K]^+$	C48H84NO8PK	872.556614	872.55677	0.179
CerP(36:1)	$[M-H]^-$	C36H72NO6P	644.50245	644.50298	0.822
SM (43:2)**	$[M+H]^+$	C48H95N2O6P	827.700052	827.70021	0.191
SM (43:2)**	$[M+Na]^+$	C48H95N2O6PNa	849.681996	849.68262	0.711
SM (43:2)**	$[M+K]^+$	C48H95N2O6PK	865.655934	865.65650	0.654

Supplementary Table 4. Detailed explanation of lipid species assignments.

List of all putative lipid species assigned to each entry in Supplementary Table 3 from the LIPID MAPS database with a 0.01 *m/z* mass tolerance. Lipid species included in Supplementary Table 3 after structural validation are indicated in bold font. Specifically, lipid species presented only in bold were identified based on MS/MS and/or by comparing the observed distributions of different adducts of the same molecule across the coronal brain tissue sections. Lipid species assignments presented in italic bold were identified solely based on mass accuracy and/or isotopic distribution. The mass error in the corresponding mass range was determined through MS/MS analysis of the nearby m/z values of other lipid species, including SHexCers.

Input Mass	Matched Mass	ppm errors	Name	Formula	Ion	Comment
848.6384	848.6375	1.002	PS 40:0	C46H90NO10P	[M+H]+	
848.6384	848.6375	1.002	IPC 40:0;O2	C46H92NO11P	[M+H-H2O]+	
848.6384	848.6375	1.002	PG 40:2	C46H87O10P	[M+NH4]+	
**848.6384**	**848.6376**	**0.884**	**HexCer(d42:2)**	**C48H91NO8K**	**[M+K]+**	**similar distribution with Na adduct ion (see SI Fig. 7)**
848.6384	848.6294	10.546	PC dO-40:4	C48H92NO6PK	[M+K]+	
848.6384	848.6480	-11.371	PC O-38:0	C46H94NO7PNa2	[M+2Na-H]+	
848.6384	848.6480	-11.371	PE O-41:0	C46H94NO7PNa2	[M+2Na-H]+	
832.6641	832.6661	-2.462	HexCer 44:4;O3	C50H91NO9	[M+H-H2O]+	
832.6641	832.6578	7.506	PC O-42:5	C50H92NO7P	[M+H-H2O]+	
**832.6641**	**832.6637**	**0.420**	**HexCer(d42:2)**	**C48H91NO8Na**	**[M+Na]+**	**similar distribution with K adduct ion (see SI Fig. 7)**
832.6641	832.6554	10.388	PC dO-40:4	C48H92NO6PNa	[M+Na]+	
864.6331	864.6324	0.798	IPC 40:1;O2	C46H90NO11P	[M+H]+	
864.6331	864.6407	-8.801	Hex2Cer 34:0;O2	C46H89NO13	[M+H]+	
864.6331	864.6324	0.798	IPC 40:0;O3	C46H92NO12P	[M+H-H2O]+	
864.6331	864.6325	0.682	HexCer 36:1;O	C48H91NO9K	[M+K]+	
**864.6331**	**864.6325**	**0.682**	**HexCer(t42:2)**	**C48H91NO9K**	**[M+K]+**	**MS/MS**
864.6331	864.6243	10.166	PC O-40:3	C48H92NO7PK	[M+K]+	
864.6331	864.6300	3.574	PS 41:2	C47H88NO10PLi	[M+Li]+	
848.6591	848.6528	7.400	PC O-42:6	C50H90NO7P	[M+H]+	
848.6591	848.6528	7.400	PC 42:4	C50H92NO8P	[M+H-H2O]+	
848.6591	848.6586	0.566	HexCer 36:1;O	C48H91NO9Na	[M+Na]+	
**848.6591**	**848.6586**	**0.566**	**HexCer(t42:2)**	**C48H91NO9Na**	**[M+Na]+**	**similar distribution with K adduct ion (see SI Fig. 7)**
848.6591	848.6504	10.228	PC O-40:3	C48H92NO7PNa	[M+Na]+	
850.6526	850.6532	-0.764	PG 40:1	C46H89O10P	[M+NH4]+	

**850.6526**	**850.6533**	**-0.882**	**HexCer(d42:1)**	**C48H93NO8K**	**[M+K]+**	**similar distribution with Na adduct ion (see SI Fig. 7)**
834.6787	834.6735	6.182	PC O-42:4	C50H94NO7P	[M+H-H2O]+	
**834.6787**	**834.6793**	**-0.767**	**HexCer(d42:1)**	**C48H93NO8Na**	**[M+Na]+**	
866.6480	866.6481	-0.150	IPC 40:0;O2	C46H92NO11P	[M+H]+	
**866.6480**	**866.6482**	**-0.265**	**HexCer(t42:1)**	**C48H93NO9K**	**[M+K]+**	**similar distribution with Na adduct ion (see SI Fig. 7)**
866.6480	866.6399	9.312	PC O-40:2	C48H94NO7PK	[M+K]+	
866.6480	866.6457	2.619	PS 41:1	C47H90NO10PLi	[M+Li]+	
850.6735	850.6767	-3.797	HexCer 44:4;O3	C50H91NO9	[M+H]+	
850.6735	850.6684	5.960	PC O-42:5	C50H92NO7P	[M+H]+	
850.6735	850.6684	5.960	PC 42:3	C50H94NO8P	[M+H-H2O]+	
**850.6735**	**850.6742**	**-0.858**	**HexCer(t42:1)**	**C48H93NO9Na**	**[M+Na]+**	**similar distribution with K adduct ion (see SI Fig. 7)**
850.6735	850.6660	8.781	PC O-40:2	C48H94NO7PNa	[M+Na]+	
**813.6844**	**813.6844**	**0.012**	**SM (42:2)**	**C47H93N2O6P**	**[M+H]+**	**MS/MS**
813.6844	813.6943	-12.154	TG 47:1	C50H94O6Na	[M+Na]+	
813.6844	813.6926	-10.065	HexCer 41:2;O2	C47H89NO8	[M+NH4]+	
813.6844	813.6919	-9.205	PG dO-40:0	C46H95O8PLi	[M+Li]+	
**835.6661**	**835.6663**	**-0.263**	**SM (42:2)**	**C47H93N2O6PNa**	**[M+Na]+**	**MS/MS**
851.6403	851.6484	-9.487	PS 39:0	C45H88NO10P	[M+NH4]+	
**851.6403**	**851.6403**	**0.023**	**SM(42:2)**	**C47H93N2O6PK**	**[M+K]+**	**MS/MS**
851.6403	851.6476	-8.548	PG dO-40:0	C46H95O8PNa2	[M+2Na-H]+	
851.6403	851.6348	6.482	PG 41:2	C47H89O10PLi	[M+Li]+	
**815.7001**	**815.7001**	**0.071**	**SM(42:2)**	**C47H95N2O6P**	**[M+H]+**	**MS/MS**
815.7001	815.7099	-12.002	TG 47:0	C50H96O6Na	[M+Na]+	
815.7001	815.7083	-10.040	HexCer 41:1;O2	C47H91NO8	[M+NH4]+	
837.6817	837.6820	-0.334	SM 42:1;O2	C47H95N2O6PNa	[M+Na]+	
**853.6559**	**853.6559**	**0.012**	**SM(42:2)**	**C47H95N2O6PK**	**[M+K]+**	**MS/MS**
853.6559	853.6504	6.455	PG 41:1	C47H91O10PLi	[M+Li]+	
**788.6164**	**788.6164**	**0.013**	**PC 36:1**	**C44H86NO8P**	**[M+H]+**	**MS/MS**
788.6164	788.6164	0.013	PE 39:1	C44H86NO8P	[M+H]+	
788.6164	788.6246	-10.385	HexCer 38:1;O4	C44H85NO10	[M+H]+	
788.6164	788.6164	0.013	PS O-38:0	C44H88NO9P	[M+H-H2O]+	

788.6164	788.6164	0.013	PA 41:2	C44H83O8P	[M+NH4]+	
788.6164	788.6140	3.056	PC O-37:4	C45H84NO7PLi	[M+Li]+	
788.6164	788.6140	3.056	PE O-40:4	C45H84NO7PLi	[M+Li]+	
788.6164	788.6222	-7.342	HexCer 39:3;O3	C45H83NO9Li	[M+Li]+	
810.5989	810.6007	-2.171	PC 38:4	C46H84NO8P	[M+H]+	
810.5989	810.6007	-2.171	PE 41:4	C46H84NO8P	[M+H]+	
810.5989	810.6007	-2.171	PS O-40:3	C46H86NO9P	[M+H-H2O]+	
**810.5989**	**810.5983**	**0.790**	**PC 36:1**	**C44H86NO8PNa**	**[M+Na]+**	**MS/MS**
810.5989	810.5983	0.790	PE 39:1	C44H86NO8PNa	[M+Na]+	
810.5989	810.6066	-9.450	HexCer 38:1;O4	C44H85NO10Na	[M+Na]+	
810.5989	810.5983	0.790	PE O-42:7	C47H82NO7PLi	[M+Li]+	
826.5729	826.5804	-9.086	IPC 36:0;O3	C42H84NO12P	[M+H]+	
826.5729	826.5745	-1.948	PE 44:8	C49H82NO8P	[M+H-H2O]+	
826.5729	826.5721	0.956	PE O-42:7	C47H82NO7PNa	[M+Na]+	
826.5729	826.5804	-9.086	PI O-33:1	C42H81O12P	[M+NH4]+	
**826.5729**	**826.5723**	**0.714**	**PC 36:1**	**C44H86NO8PK**	**[M+K]+**	**MS/MS**
826.5729	826.5723	0.714	PE 39:1	C44H86NO8PK	[M+K]+	
826.5729	826.5805	-9.207	HexCer 38:1;O4	C44H85NO10K	[M+K]+	
826.5729	826.5697	3.859	PC O-37:4	C45H84NO7PNa2	[M+2Na-H]+	
826.5729	826.5697	3.859	PE O-40:4	C45H84NO7PNa2	[M+2Na-H]+	
826.5729	826.5779	-6.061	HexCer 39:3;O3	C45H83NO9Na2	[M+2Na-H]+	
826.5729	826.5804	-9.086	DGCC 40:10	C50H77NO8Li	[M+Li]+	
**834.6013**	**834.6007**	**0.683**	**PC 40:6**	**C48H84NO8P**	**[M+H]+**	**MS/MS**
834.6013	834.6007	0.683	PE 43:6	C48H84NO8P	[M+H]+	
834.6013	834.5937	9.070	Hex2Cer 32:1;O2	C44H83NO13	[M+H]+	
834.6013	834.6007	0.683	PS O-42:5	C48H86NO9P	[M+H-H2O]+	
834.6013	834.5983	3.559	PC 38:3	C46H86NO8PNa	[M+Na]+	
834.6013	834.5983	3.559	PE 41:3	C46H86NO8PNa	[M+Na]+	
834.6013	834.6042	-3.511	HexCer 38:0;O4	C44H87NO10Na2	[M+2Na-H]+	
834.6013	834.5959	6.434	PC 36:0	C44H88NO8PNa2	[M+2Na-H]+	
834.6013	834.5959	6.434	PE 39:0	C44H88NO8PNa2	[M+2Na-H]+	
856.5828	856.5851	-2.720	PC 42:9	C50H82NO8P	[M+H]+	
**856.5828**	**856.5827**	**0.082**	**PC 40:6**	**C48H84NO8PNa**	**[M+Na]+**	**MS/MS**
856.5828	856.5827	0.082	PE 43:6	C48H84NO8PNa	[M+Na]+	
856.5828	856.5757	8.254	Hex2Cer 32:1;O2	C44H83NO13Na	[M+Na]+	
856.5828	856.5910	-9.608	PI 34:0	C43H83O13P	[M+NH4]+	
856.5828	856.5828	-0.035	PS O-39:1	C45H88NO9PK	[M+K]+	
856.5828	856.5803	2.884	PC 38:3	C46H86NO8PNa2	[M+2Na-H]+	
856.5828	856.5803	2.884	PE 41:3	C46H86NO8PNa2	[M+2Na-H]+	
**872.5568**	**872.5566**	**0.195**	**PC 40:6**	**C48H84NO8PK**	**[M+K]+**	**MS/MS**
872.5568	872.5566	0.195	PE 43:6	C48H84NO8PK	[M+K]+	

872.5568	872.5496	8.217	Hex2Cer 32:1;O2	C44H83NO13K	[M+K]+	
865.6565	865.6641	-8.779	PS 40:0	C46H90NO10P	[M+NH4]+	
865.6565	***865.6559***	***0.693***	***SM 43:2***	***C48H95N2O6PK***	***[M+K]+***	***mass accuracy***
865.6565	865.6504	7.047	PG 42:2	C48H91O10PLi	[M+Li]+	
849.6826	***849.6820***	***0.706***	***SM 43:2***	***C48H95N2O6PNa***	***[M+Na]+***	***mass accuracy***
849.6826	849.6919	-10.945	TG 48:1	C51H96O6Na2	[M+2Na-H]+	
827.7002	***827.7000***	***0.242***	***SM 43:2***	***C48H95N2O6P***	***[M+H]+***	***mass accuracy***
827.7002	827.7000	0.242	PC dO-40:4	C48H92NO6P	[M+NH4]+	
827.7002	827.7083	-9.786	HexCer 42:2;O2	C48H91NO8	[M+NH4]+	
720.4366	720.4377	-1.596	PE 32:3	C37H68NO8P	[M+Cl]-	
720.4366	720.4457	-12.700	PC 27:2;O	C35H66NO9P	[M+Formate]-	
720.4366	***720.4362***	***0.476***	***SHexCer(d30:2)***	***C36H67NO11S***	***[M-H]-***	***mass accuracy***
736.4312	736.4406	-12.723	PC 27:2;O2	C35H66NO10P	[M+Formate]-	
736.4312	736.4406	-12.723	PS 29:1	C35H66NO10P	[M+Formate]-	
736.4312	736.4406	-12.723	PS 28:1	C34H64NO10P	[M+OAc]-	
736.4312	***736.4311***	***0.162***	***SHexCer(t30:2)***	***C36H67NO12S***	***[M-H]-***	***mass accuracy***
750.4834	750.4927	-12.445	IPC 32:1;O2	C38H74NO11P	[M-H]-	
750.4834	750.4846	-1.652	PC 31:2	C39H74NO8P	[M+Cl]-	
750.4834	750.4846	-1.652	PE 34:2	C39H74NO8P	[M+Cl]-	
750.4834	750.4927	-12.445	PS O-31:1	C37H72NO9P	[M+Formate]-	
750.4834	750.4927	-12.445	PS O-30:1	C36H70NO9P	[M+OAc]-	
750.4834	***750.4832***	***0.284***	***SHexCer(d32:1)***	***C38H73NO11S***	***[M-H]-***	***mass accuracy***
766.4786	766.4795	-1.226	PS O-33:2	C39H74NO9P	[M+Cl]-	
766.4786	766.4876	-11.794	PS 31:0	C37H72NO10P	[M+Formate]-	
766.4786	766.4876	-11.794	PS 30:0	C36H70NO10P	[M+OAc]-	
766.4786	***766.4781***	***0.637***	***SHexCer(t32:1)***	***C38H73NO12S***	***[M-H]-***	***mass accuracy***
748.4677	748.4690	-1.697	PC 31:3	C39H72NO8P	[M+Cl]-	
748.4677	748.4690	-1.697	PE 34:3	C39H72NO8P	[M+Cl]-	
748.4677	748.4770	-12.385	PS O-31:2	C37H70NO9P	[M+Formate]-	
748.4677	748.4770	-12.385	PS O-30:2	C36H68NO9P	[M+OAc]-	
748.4677	***748.4675***	***0.298***	***SHexCer(d32:2)***	***C38H71NO11S***	***[M-H]-***	***mass accuracy***
764.4631	764.4639	-1.099	PS O-33:3	C39H72NO9P	[M+Cl]-	
764.4631	764.4719	-11.564	PS 31:1	C37H70NO10P	[M+Formate]-	
764.4631	764.4719	-11.564	PC 28:2;O2	C36H68NO10P	[M+OAc]-	
764.4631	764.4719	-11.564	PS 30:1	C36H68NO10P	[M+OAc]-	
764.4631	***764.4624***	***0.836***	***SHexCer(t32:2)***	***C38H71NO12S***	***[M-H]-***	***mass accuracy***

776.4990	776.5003	-1.648	PC 33:3	C41H76NO8P	[M+Cl]-	
776.4990	776.5003	-1.648	PE 36:3	C41H76NO8P	[M+Cl]-	
776.4990	776.5083	-11.951	PS O-33:2	C39H74NO9P	[M+Formate]-	
776.4990	776.5083	-11.951	PS O-32:2	C38H72NO9P	[M+OAc]-	
776.4990	***776.4988***	***0.274***	***SHexCer(d34:2)***	***C40H75NO11S***	***[M-H]-***	***mass accuracy***
792.4941	792.4952	-1.413	PS O-35:3	C41H76NO9P	[M+Cl]-	
792.4941	792.5032	-11.508	PS 33:1	C39H74NO10P	[M+Formate]-	
792.4941	792.5032	-11.508	PS 32:1	C38H72NO10P	[M+OAc]-	
792.4941	***792.4937***	***0.452***	***SHexCer(d34:2)***	***C40H75NO12S***	***[M-H]-***	***mass accuracy***
804.5309	804.5316	-0.870	PC 35:3	C43H80NO8P	[M+Cl]-	
804.5309	804.5316	-0.870	PE 38:3	C43H80NO8P	[M+Cl]-	
804.5309	804.5396	-10.814	PS O-35:2	C41H78NO9P	[M+Formate]-	
804.5309	804.5396	-10.814	PS O-34:2	C40H76NO9P	[M+OAc]-	
804.5309	***804.5301***	***0.986***	***SHexCer(d36:2)***	***C42H79NO11S***	***[M-H]-***	***mass accuracy-isotopic distribution***
820.5256	820.5265	-1.158	PS O-37:3	C43H80NO9P	[M+Cl]-	
820.5256	820.5345	-10.908	PS 35:1	C41H78NO10P	[M+Formate]-	
820.5256	820.5345	-10.908	PS 34:1	C40H76NO10P	[M+OAc]-	
820.5256	***820.5250***	***0.643***	***SHexCer(t36:2)***	***C42H79NO12S***	***[M-H]-***	***mass accuracy***
**806.5465**	**806.5458**	**0.880**	**SHexCer(d36:1)**	**C42H81NO11S**	**[M-H]-**	**MSMS head group**
806.5465	806.5553	-10.898	IPC 36:1;O2	C42H82NO11P	[M-H]-	
806.5465	806.5472	-0.855	PC 35:2	C43H82NO8P	[M+Cl]-	
806.5465	806.5472	-0.855	PE 38:2	C43H82NO8P	[M+Cl]-	
806.5465	806.5472	-0.855	PE-NMe2 36:2	C43H82NO8P	[M+Cl]-	
806.5465	806.5555	-11.146	HexCer 37:2;O4	C43H81NO10	[M+Cl]-	
806.5465	806.5553	-10.898	PS O-35:1	C41H80NO9P	[M+Formate]-	
806.5465	806.5553	-10.898	PS O-34:1	C40H78NO9P	[M+OAc]-	
**822.5411**	**822.5407**	**0.535**	**SHexCer(t36:1)**	**C42H81NO12S**	**[M-H]-**	**MS/MS**
822.5411	822.5421	-1.167	PS O-37:2	C43H82NO9P	[M+Cl]-	
822.5411	822.5502	-11.015	PS 35:0	C41H80NO10P	[M+Formate]-	
822.5411	822.5502	-11.015	PS 34:0	C40H78NO10P	[M+OAc]-	
832.5615	832.5629	-1.634	PC 37:3	C45H84NO8P	[M+Cl]-	
832.5615	832.5629	-1.634	PE 40:3	C45H84NO8P	[M+Cl]-	
832.5615	832.5709	-11.242	PS O-37:2	C43H82NO9P	[M+Formate]-	
832.5615	832.5709	-11.242	PS O-36:2	C42H80NO9P	[M+OAc]-	
832.5615	***832.5614***	***0.160***	***SHexCer(d38:2)***	***C44H83NO11S***	***[M-H]-***	***mass accuracy***
848.5569	848.5658	-10.477	PS 37:1	C43H82NO10P	[M+Formate]-	
848.5569	848.5658	-10.477	PT 36:1	C43H82NO10P	[M+Formate]-	
848.5569	848.5658	-10.477	PS 36:1	C42H80NO10P	[M+OAc]-	

848.5569	***848.5563***	***0.693***	***SHexCer(t38:2)***	***C44H83NO12S***	***[M-H]-***	***mass accuracy***
834.5773	834.5771	0.216	SHexCer(d38:1)	C44H85NO11S	[M-H]-	mass accuracy
834.5773	834.5785	-1.462	PC 37:2	C45H86NO8P	[M+Cl]-	
834.5773	834.5785	-1.462	PE 40:2	C45H86NO8P	[M+Cl]-	
834.5773	834.5866	-11.167	PS O-37:1	C43H84NO9P	[M+Formate]-	
834.5773	834.5866	-11.167	PS O-36:1	C42H82NO9P	[M+OAc]-	
850.5717	***850.5720***	***-0.332***	***SHexCer(t38:1)***	***C44H85NO12S***	***[M-H]-***	***mass accuracy***
850.5717	850.5734	-2.010	PS O-39:2	C45H86NO9P	[M+Cl]-	
850.5717	850.5815	-11.533	PS 37:0	C43H84NO10P	[M+Formate]-	
850.5717	850.5815	-11.533	PS 36:0	C42H82NO10P	[M+OAc]-	
860.5930	860.5942	-1.406	PC 39:3	C47H88NO8P	[M+Cl]-	
860.5930	860.5942	-1.406	PE 42:3	C47H88NO8P	[M+Cl]-	
860.5930	860.6022	-10.702	PS O-39:2	C45H86NO9P	[M+Formate]-	
860.5930	860.6022	-10.702	PS O-38:2	C44H84NO9P	[M+OAc]-	
**860.5930**	**860.5927**	**0.329**	**SHexCer(d40:2)**	**C46H87NO11S**	**[M-H]-**	**MS/MS**
876.5880	876.5971	-10.392	PS 39:1	C45H86NO10P	[M+Formate]-	
876.5880	876.5971	-10.392	PS 38:1	C44H84NO10P	[M+OAc]-	
**876.5880**	**876.5876**	**0.420**	**SHexCer(t40:2)**	**C46H87NO12S**	**[M-H]-**	**MS/MS**
**862.6079**	**862.6084**	**-0.556**	**SHexCer(d40:1)**	**C46H89NO11S**	**[M-H]-**	**MS/MS**
862.6079	862.6179	-11.569	IPC 40:1;O2	C46H90NO11P	[M-H]-	
862.6079	862.6098	-2.179	PC 39:2	C47H90NO8P	[M+Cl]-	
862.6079	862.6098	-2.179	PE 42:2	C47H90NO8P	[M+Cl]-	
862.6079	862.6179	-11.569	PS O-39:1	C45H88NO9P	[M+Formate]-	
862.6079	862.6179	-11.569	PS O-38:1	C44H86NO9P	[M+OAc]-	
**878.6034**	**878.6033**	**0.057**	**SHexCer(t40:1)**	**C46H89NO12S**	**[M-H]-**	**MS/MS**
878.6034	878.6128	-10.756	PS 39:0	C45H88NO10P	[M+Formate]-	
878.6034	878.6128	-10.756	PS 38:0	C44H86NO10P	[M+OAc]-	
890.6042	890.6128	-9.634	Am-Hex-PE O-36:2	C47H90NO12P	[M-H]-	
890.6042	890.6047	-0.539	PS O-42:3	C48H90NO9P	[M+Cl]-	
890.6042	890.6128	-9.634	PS 40:1	C46H88NO10P	[M+Formate]-	
890.6042	890.6128	-9.634	PS 39:1	C45H86NO10P	[M+OAc]-	
**890.6042**	**890.6033**	**1.064**	**SHexCer(t41:2)**	**C47H89NO12S**	**[M-H]-**	**MS/MS**
874.6080	874.6098	-2.012	PC 40:3	C48H90NO8P	[M+Cl]-	
874.6080	874.6179	-11.273	PS O-40:2	C46H88NO9P	[M+Formate]-	
874.6080	874.6179	-11.273	PS O-39:2	C45H86NO9P	[M+OAc]-	
**874.6080**	**874.6084**	**-0.362**	**SHexCer(d41:2)**	**C47H89NO11S**	**[M-H]-**	**MS/MS head group**
876.6245	876.6255	-1.164	PC 40:2	C48H92NO8P	[M+Cl]-	

876.6245	876.6255	-1.164	PE 43:2	C48H92NO8P	[M+Cl]-	
876.6245	876.6335	-10.289	PS O-40:1	C46H90NO9P	[M+Formate]-	
876.6245	876.6335	-10.289	PS O-39:1	C45H88NO9P	[M+OAc]-	
**876.6245**	**876.6240**	**0.540**	**SHexCer(d41:1)**	**C47H91NO11S**	**[M-H]-**	**MS/MS**
892.6190	892.6284	-10.508	Am-Hex-PE O-36:1	C47H92NO12P	[M-H]-	
892.6190	892.6284	-10.508	Am-Hex-PE O-36:2	C47H92NO12P	[M-H]-	
892.6190	892.6204	-1.546	PS O-42:2	C48H92NO9P	[M+Cl]-	
892.6190	892.6284	-10.508	PS 40:0	C46H90NO10P	[M+Formate]-	
892.6190	892.6284	-10.508	PS 39:0	C45H88NO10P	[M+OAc]-	
**892.6190**	**892.6189**	**0.110**	**SHexCer(t41:1)**	**C47H91NO12S**	**[M-H]-**	**MS/MS**
902.6040	902.6128	-9.727	PS 41:2	C47H88NO10P	[M+Formate]-	
902.6040	902.6128	-9.727	PS 40:2	C46H86NO10P	[M+OAc]-	
902.6040	***902.6033***	***0.829***	***SHexCer(t42:3)***	***C48H89NO12S***	***[M-H]-***	***mass accuracy***
886.6091	886.6098	-0.778	PC 41:4	C49H90NO8P	[M+Cl]-	
886.6091	886.6098	-0.778	PE 44:4	C49H90NO8P	[M+Cl]-	
886.6091	886.6179	-9.914	PS O-40:3	C46H86NO9P	[M+OAc]-	
**886.6091**	**886.6084**	**0.849**	**SHexCer(d42:3)**	**C48H89NO11S**	**[M-H]-**	**MS/MS head group**
**888.6240**	**888.6240**	**0.023**	**SHexCer(d42:2)**	**C48H91NO11S**	**[M-H]-**	**MS/MS**
888.6240	888.6255	-1.665	PC 41:3	C49H92NO8P	[M+Cl]-	
888.6240	888.6255	-1.665	PE 44:3	C49H92NO8P	[M+Cl]-	
888.6240	888.6335	-10.668	PS O-40:2	C46H88NO9P	[M+OAc]-	
**904.6198**	**904.6189**	**0.984**	**SHexCer(t42:2)**	**C48H91NO12S**	**[M-H]-**	**MS/MS**
904.6198	904.6286	-9.739	HexCer 43:3;O5	C49H91NO11	[M+Cl]-	
904.6198	904.6284	-9.518	PS 41:1	C47H90NO10P	[M+Formate]-	
904.6198	904.6284	-9.518	PS 40:1	C46H88NO10P	[M+OAc]-	
**890.6400**	**890.6397**	**0.281**	**SHexCer(d42:1)**	**C48H93NO11S**	**[M-H]-**	**MS/MS**
890.6400	890.6411	-1.291	PC 41:2	C49H94NO8P	[M+Cl]-	
890.6400	890.6411	-1.291	PE 44:2	C49H94NO8P	[M+Cl]-	
890.6400	890.6492	-10.386	PS O-41:1	C47H92NO9P	[M+Formate]-	
890.6400	890.6492	-10.386	PS O-40:1	C46H90NO9P	[M+OAc]-	
**906.6346**	**906.6346**	**0.044**	**SHexCer(d42:1)**	**C48H93NO12S**	**[M-H]-**	**MS/MS**
906.6346	906.6443	-10.655	HexCer 43:2;O5	C49H93NO11	[M+Cl]-	
906.6346	906.6441	-10.434	PS 41:0	C47H92NO10P	[M+Formate]-	
906.6346	906.6441	-10.434	PS 40:0	C46H90NO10P	[M+OAc]-	
902.6400	902.6411	-1.274	PC 42:3	C50H94NO8P	[M+Cl]-	
902.6400	902.6492	-10.248	PS O-42:2	C48H92NO9P	[M+Formate]-	
**902.6400**	**902.6397**	**0.323**	**SHexCer(d43:2)**	**C49H93NO11S**	**[M-H]-**	**MS/MS**

918.6355	918.6441	-9.405	PS 42:1	C48H92NO10P	[M+Formate]-	
918.6355	918.6441	-9.405	PS 41:1	C47H90NO10P	[M+OAc]-	
**918.6355**	**918.6346**	**0.967**	**SHexCer(t43:2)**	**C49H93NO12S**	**[M-H]-**	**MS/MS**
914.6408	914.6411	-0.328	PC 43:4	C51H94NO8P	[M+Cl]-	
914.6408	914.6492	-9.184	PS O-42:3	C48H90NO9P	[M+OAc]-	
914.6408	***914.6397***	***1.249***	***SHexCer(d44:3)***	***C50H93NO11S***	***[M-H]-***	***mass accuracy-isotopic distribution***
930.6360	930.6441	-8.693	PS 43:2	C49H92NO10P	[M+Formate]-	
930.6360	930.6441	-8.693	PS 42:2	C48H90NO10P	[M+OAc]-	
**930.6360**	**930.6346**	**1.545**	**SHexCer(t44:3)**	**C50H93NO12S**	**[M-H]-**	**MS/MS**
916.6555	916.6648	-10.102	PS O-42:2	C48H92NO9P	[M+OAc]-	
**916.6555**	**916.6553**	**0.253**	**SHexCer(d44:2)**	**C50H95NO11S**	**[M-H]-**	**MS/MS**
932.6509	932.6597	-9.425	PS 43:1	C49H94NO10P	[M+Formate]-	
932.6509	932.6597	-9.425	PS 42:1	C48H92NO10P	[M+OAc]-	
**932.6509**	**932.6502**	**0.738**	**SHexCer(t44:2)**	**C50H95NO12S**	**[M-H]-**	**MS/MS**
918.6716	918.6724	-0.903	PC 43:2	C51H98NO8P	[M+Cl]-	
918.6716	918.6805	-9.720	PS O-42:1	C48H94NO9P	[M+OAc]-	
918.6716	***918.6710***	***0.666***	***SHexCer(d44:1)***	***C50H97NO11S***	***[M-H]-***	***mass accuracy***
934.6667	934.6754	-9.297	PS 43:0	C49H96NO10P	[M+Formate]-	
934.6667	934.6754	-9.297	PS 42:0	C48H94NO10P	[M+OAc]-	
**934.6667**	**934.6659**	**0.897**	**SHexCer(t44:1)**	**C50H97NO12S**	**[M-H]-**	**MS/MS**
920.6510	920.6597	-9.504	PS 42:0	C48H94NO10P	[M+Formate]-	
920.6510	920.6597	-9.504	PS 41:0	C47H92NO10P	[M+OAc]-	
**920.6510**	**920.6502**	**0.791**	**SHexCer(d43:1)**	**C49H95NO12S**	**[M-H]-**	**MS/MS**
**1207.7685**	**1207.7685**	**0.008**	**NeuAcHex2Cer 38:1;O2/GM3(38:1)**	**C61H112N2O21**	**[M-H]-**	**MS/MS**
**1572.9008**	**1572.9007**	**0.083**	**Hex(3)-HexNAc-NeuAc-Cer 38:1;O2/GM1(38:1)**	**C75H135N3O31**	**[M-H]-**	**MS/MS**
1572.9008	1572.9007	0.083	NeuAc2Hex2Cer 40:1;O2	C74H133N3O29	[M+Formate]-	
**1544.8705**	**1544.8694**	**0.731**	**Hex(3)-HexNAc-NeuAc-Cer 36:1;O2/GM1(36:1)**	**C73H131N3O31**	**[M-H]-**	**MS/MS**
1544.8705	1544.8694	0.731	NeuAc2Hex2Cer 38:1;O2	C72H129N3O29	[M+Formate]-	
**1382.8155**	**1382.8166**	**-0.810**	**Hex(2)-HexNAc-NeuAc-Cer 36:1;O2/GM2(36:1)**	**C67H121N3O26**	**[M-H]-**	**MS/MS**

**1179.7365**	**1179.7372**	**-0.559**	**NeuAcHex2Cer 36:1;O2/GM3(36:1)**	**C59H108N2O21**	**[M-H]-**	**MS/MS**
1901.9503	1901.9521	-0.973	Hex(3)-HexNAc(2)-Fuc-NeuAc-Cer 34:1;O2	C85H150N4O40	[M+Cl]-	
**1901.9503**	**1901.9520**	**-0.907**	**GD1+K-2H(38:1)**	**C86H152N4O39K**	**[M+K-2H]-**	**MS/MS**
**1873.9207**	**1873.9217**	**-0.530**	**GD1+K-2H(38:1)**	**C86H152N4O39Na**	**[M+K-2H]-**	**MS/MS**
**1835.9659**	**1835.9648**	**0.621**	**Hex(3)-HexNAc-NeuAc(2)-Cer 36:1;O2/GD1(36:1)**	**C84H148N4O39**	**[M-H]-**	**MS/MS**
1835.9659	1835.9648	0.621	Hex(2)-NeuAc(3)-Cer 38:1;O2	C83H146N4O37	[M+Formate]-	
**1863.9958**	**1863.9961**	**-0.150**	**Hex(3)-HexNAc-NeuAc(2)-Cer 38:1;O2/GD1(38:1)**	**C86H152N4O39**	**[M-H]-**	**MS/MS**
1863.9958	1863.9961	-0.150	Hex(2)-NeuAc(3)-Cer 40:1;O2	C85H150N4O37	[M+Formate]-	
**788.5455**	**788.5447**	**0.951**	**PS 36:1**	**C42H80NO10P**	**[M-H]-**	**MS/MS**
788.5455	788.5449	0.697	HexCer 37:3;O3	C43H79NO9	[M+Cl]-	
788.5455	788.5367	11.097	PC O-35:4	C43H80NO7P	[M+Cl]-	
788.5455	788.5367	11.097	PE O-38:4	C43H80NO7P	[M+Cl]-	
788.5455	788.5447	0.951	PC 33:2	C41H78NO8P	[M+Formate]-	
788.5455	788.5447	0.951	PE 36:2	C41H78NO8P	[M+Formate]-	
788.5455	788.5530	-9.574	HexCer 35:2;O4	C41H77NO10	[M+Formate]-	
788.5455	788.5447	0.951	PC 32:2	C40H76NO8P	[M+OAc]-	
788.5455	788.5447	0.951	PE 35:2	C40H76NO8P	[M+OAc]-	
**834.5295**	**834.5291**	**0.491**	**PS 40:6**	**C46H78NO10P**	**[M-H]-**	**MS/MS**
834.5295	834.5291	0.491	PC 37:7	C45H76NO8P	[M+Formate]-	
834.5295	834.5291	0.491	PE 40:7	C45H76NO8P	[M+Formate]-	
834.5295	834.5291	0.491	PC 36:7	C44H74NO8P	[M+OAc]-	
834.5295	834.5291	0.491	PE 39:7	C44H74NO8P	[M+OAc]-	
**762.5083**	**762.5079**	**0.551**	**PE 38:6**	**C43H74NO8P**	**[M-H]-**	**MS/MS**
762.5083	762.5079	0.551	PE O-38:7;O	C43H74NO8P	[M-H]-	
762.5083	762.5079	0.551	PC 36:6	C44H76NO8P	[M-CH3]-	
**790.5396**	**790.5392**	**0.468**	**PE 40:6**	**C45H78NO8P**	**[M-H]-**	**MS/MS**
790.5396	790.5392	0.468	PE O-40:7;O	C45H78NO8P	[M-H]-	
790.5396	790.5392	0.468	PE dO-38:8	C43H74NO6P	[M+OAc]-	
790.5396	790.5392	0.468	PC 38:6	C46H80NO8P	[M-CH3]-	
**857.5182**	**857.5186**	**-0.455**	**PI 36:4**	**C45H79O13P**	**[M-H]-**	**MS/MS**
857.5182	857.5105	8.991	PG 40:6	C46H79O10P	[M+Cl]-	

885.5503	885.5499	0.497	Glc-GP 38:4	C47H83O13P	[M-H]-	
**885.5503**	**885.5499**	**0.497**	**PI 38:4**	**C47H83O13P**	**[M-H]-**	**MS/MS**
885.5503	885.5581	-8.763	DGDG 32:3	C47H82O15	[M-H]-	
885.5503	885.5534	-3.455	SQDG 36:0	C45H86O12S	[M+Cl]-	
885.5503	885.5418	9.644	PG 42:6	C48H83O10P	[M+Cl]-	
**909.5508**	**909.5499**	**0.968**	**PI 40:6**	**C49H83O13P**	**[M-H]-**	**MS/MS**
909.5508	909.5418	9.873	PG 44:8	C50H83O10P	[M+Cl]-	
718.5397	718.5392	0.640	PE 34:0	C39H78NO8P	[M-H]-	
**718.5397**	**718.5392**	**0.640**	**PE-NMe2 32:0**	**C39H78NO8P**	**[M-H]-**	**MS/MS**
718.5397	718.5394	0.362	DGTA 30:0	C40H77NO7	[M+Cl]-	
718.5397	718.5392	0.640	CerP 38:1;O2	C38H76NO6P	[M+Formate]-	
718.5397	718.5475	-10.911	HexCer 32:0;O2	C38H75NO8	[M+Formate]-	
718.5397	718.5392	0.640	PC 32:0	C40H80NO8P	[M-CH3]-	
**766.5398**	**766.5392**	**0.744**	**PE 38:4**	**C43H78NO8P**	**[M-H]-**	**MS/MS**
766.5398	766.5392	0.744	PE O-38:5;O	C43H78NO8P	[M-H]-	
766.5398	766.5392	0.744	PC 36:4	C44H80NO8P	[M-CH3]-	
**774.5448**	**774.5443**	**0.671**	**PE O-40:7/PE-P-(40:6)**	**C45H78NO7P**	**[M-H]-**	**MS/MS**
774.5448	774.5443	0.671	PC O-38:7	C46H80NO7P	[M-CH3]-	
644.5030	***644.5025***	***0.745***	***CerP 36:1;O2***	***C36H72NO6P***	***[M-H]-***	***mass accuracy***

References

1 Hsu. F. F. & Turk. J. Studies on sulfatides by quadrupole ion-trap mass spectrometry with electrospray ionization: structural characterization and the fragmentation processes that include an unusual internal galactose residue loss and the classical charge-remote fragmentation. *J. Am. Soc. Mass Spectrom.* **15**. 536-546 (2004). https://doi.org:10.1016/j.jasms.2003.12.007

2 Colsch. B.. Jackson. S. N.. Dutta. S. & Woods. A. S. Molecular Microscopy of Brain Gangliosides: Illustrating their Distribution in Hippocampal Cell Layers. *ACS Chem. Neurosci.* **2**. 213-222 (2011). https://doi.org:10.1021/cn100096h
